# Genetic Distinctiveness of Rye *In situ* Accessions from Portugal Unveils a New Hotspot of Unexplored Genetic Resources

**DOI:** 10.3389/fpls.2016.01334

**Published:** 2016-08-31

**Authors:** Filipa Monteiro, Patrícia Vidigal, André B. Barros, Ana Monteiro, Hugo R. Oliveira, Wanda Viegas

**Affiliations:** ^1^Linking Landscape, Environment, Agriculture and Food, Instituto Superior de Agronomia, Universidade de LisboaLisboa, Portugal; ^2^Colégio F3 Food, Farming and Forestry, Universidade de LisboaLisboa, Portugal; ^3^Plant Biology/Centro de Investigação em Biodiversidade e Recursos Genéticos, Universidade do PortoPorto, Portugal; ^4^Faculty of Life Sciences, Manchester Institute of Biotechnology, University of ManchesterManchester, UK

**Keywords:** *Secale cereale*, microsatellite, *in situ* conservation, population structure, genetic pool

## Abstract

Rye (*Secale cereale* L.) is a cereal crop of major importance in many parts of Europe and rye breeders are presently very concerned with the restrict pool of rye genetic resources available. Such narrowing of rye genetic diversity results from the presence of “Petkus” pool in most modern rye varieties as well as “Petkus” × “Carsten” heterotic pool in hybrid rye breeding programs. Previous studies on rye's genetic diversity revealed moreover a common genetic background on landraces (*ex situ*) and cultivars, regardless of breeding level or geographical origin. Thus evaluation of *in situ* populations is of utmost importance to unveil “on farm” diversity, which is largely undervalued. Here, we perform the first comprehensive assessment of rye's genetic diversity and population structuring using cultivars, *ex situ* landraces along a comprehensive sampling of *in situ* accessions from Portugal, through a molecular-directed analysis using SSRs markers. Rye genetic diversity and population structure analysis does not present any geographical trend but disclosed marked differences between genetic backgrounds of *in situ* accessions and those of cultivars/*ex situ* collections. Such genetic distinctiveness of *in situ* accessions highlights their unexplored potential as new genetic resources, which can be used to boost rye breeding strategies and the production of new varieties. Overall, our study successfully demonstrates the high prospective impact of comparing genetic diversity and structure of cultivars, *ex situ*, and *in situ* samples in ascertaining the status of plant genetic resources (PGR).

## Introduction

Rye (*Secale cereale* L.) belongs to the Triticeae tribe, along with other economically important cereals such as wheat and barley. Much controversy about the taxonomy of the *Secale* genus remains, despite the large number of studies performed (e.g., Roshevitz, 1947; Delipavlov, 1962; Kobyljanskij, 1975; Sencer and Hawkes, 1980; Frederiksen and Petersen, 1998; Chikmawati et al., 2005). According to taxonomic system adopted by the American Germplasm Resources Information Network (GRIN, http://www.ars-grin.gov), *Secale* comprises four species, namely *S. cereale* L., *S. sylvestre* Host, *S. vavilovii* Grossh., and *S. strictum* (Presl.) Presl. (syn. *S. montanum* Guss). Within *S. cereale*, eight subspecies are recognized: *afghanicum* (Vavilov) K. Hammer, *ancestrale* Zhuk., *dighoricum* Vavilov, forma unranked *rigidum* Antropov and Antropova, *segetale* Zhuk., *tetraploidum* Kobyl, *tsitsinii* Kobyl and *cereale* L. (the only cultivated). Within *S. strictum*, there are five subspecies: *africanum* (Stapf) K. Hammer, *anatolicum* (Boiss.) K. Hammer, *ciliatoglume* (Boiss.) K. Hammer, *kuprijanovii* (Grossh.) K. Hammer and *strictum* (syn. *S. montanum* Guss.; GRIN, http://www.ars-grin.gov/cgi-bin/npgs/html/splist.pl?11022, 20th July 2016).

Rye is commonly grown in Eastern and Northern Europe, mainly for the production of bread, alcohol, and animal feed (Evans, 1995). In contrast to most grain crops that are self-pollinating, rye is a cross-pollinating cereal, and such outbreeding nature results in a high intraspecific diversity (Schlegel, 2014). Additionally, rye harbors a broad tolerance to biotic and abiotic stress, absent in other temperate cereals (Rizvi and Scoles, 2014). Therefore, this crop also had a major importance on plant breeding strategies both through the production of the synthetic hybrid Triticale (x *Triticosecale* Wittmack) as well as through the introgression of rye chromatin in wheat varieties, particularly by the short arm of chromosome 1R (1RS), as a source of genes for agronomic and resistant improvement (Baum and Appels, 1991). Due to its higher ability to grow in poor soils and under greater adverse conditions than other cereals, rye is an economically important cover crop in Northern Europe and other rye-growing countries (Vaughan and Geissler, 2009). Rye culture is of marked importance in the northern region of Portugal where local farmers cultivate the same rye population for several centuries under a subsistence agricultural system in small areas for both food and feed. Traditional rye bread baking is an important share of both diet and cultural heritage not only in Portugal but also in other rye-producing countries. Until the middle of last century there was genetic exchanges as a result of transhumance linking the territory to remote and dispersed regions, mainly by pastoralism that have worked as ecological corridors from valleys to the mountainous areas. Considering, that 80% of Portuguese soils are acidic (Almeida, 1955), it has been shown that Northern rye populations display not only high genetic diversity on storage proteins (Ribeiro et al., 2012) but also on aluminum tolerance (Matos et al., 2001), probably responsible for rye maintenance in the regional agricultural system. Portugal has a wealth of rye germplasm with about 769 accessions conserved in several institutions (Bettencourt and Carnide, 1998); with many other local accessions yet to be preserved/identified. According to FAO (Food and Agricultural Organization of the United Nations, 2016), in Portugal, there was a 33% decline of total rye harvest area from 2004 to 2014 as a result of traditional agricultural abandonment, which poses a huge threat toward local rye landraces. There is therefore some urgency to characterize and evaluate landraces maintained on “on farm” conditions, in order to develop proper measures for *in situ* conservation and made available for utilization.

While much of the world's rye harvest is based on modern high-yield varieties, traditional varieties grown locally have great importance as a resource for future crop improvement. Such local landraces may represent an intermediate stage of domestication between a wild ancestor and modern varieties, being important reservoirs of agronomically important genes. Landrace can be defined following (Camacho-Villa et al., 2005), as: “a dynamic population of a cultivated plant that has historic origin, distinct identity and lacks formal crop improvement, as well as often being genetically diverse, locally adapted and associated with traditional farming systems.” Furthermore, landraces can be separated in *ex situ* and *in situ* collections: the former being those detained in gene banks or botanical gardens which represent a comprehensive snapshot of the genetic diversity at a given time and place (Greene et al., 2014); while *in situ* allows adaptive evolutionary processes to continue shaping genetic diversity under farmer management. In plant genetic resources (PGR) conservation, it has long been recognized that effective strategies need to integrate *in situ* and *ex situ* approaches (Greene et al., 2014). Studies reported the complementary source of genetic variation between *in situ* and *ex situ* collections, as some of the alleles may have been lost *in situ* (e.g., Jensen et al., 2012) or *ex situ* (e.g., Li et al., 2005). Indeed, simple sequence repeat (SSR) data from bean (*Phaseolus vulgare* L.) landraces conserved *ex situ* and *in situ* indicated significant genetic differentiation in *ex situ* subpopulations as well as loss of alleles, gain of new alleles, and reduction of rare alleles with an increase of common alleles (Negri and Tiranti, 2010). In most crops, landraces usually display higher genetic diversity than breeding cultivars, due to the genetic bottleneck and selective effect associated to its improvement (Meyer and Purugganan, 2013). In fact, the narrowing of the genetic pool of modern crop varieties has become an increasing concern also for rye breeders (Fischer et al., 2010). “Petkus” was one of the leading cultivars in the twentieth century from which many of the open pollinated varieties (OPVs) were selected or include “Petkus” in their ancestry (Hepting, 1978; Miedaner, 1997; Fischer et al., 2010). The two genetic pools “Carsten” and “Petkus” were previously identified as the most promising heterotic pattern (Hepting, 1978), and from then onwards, hybrid rye breeding, a breeding system used for cross-fertilized crops, was and still is based on the “Petkus” × “Carsten” heterotic pattern (Geiger and Miedaner, 2009). Recent evidences point out for a genetic narrowing of “Carsten” pool (Fischer et al., 2010). Also, considering that the steadily improved “Petkus” was the parental ancestor of many OPVs, the probability of finding genetically diverse populations from “Petkus” pool is significantly reduced. As such, assessment of new genetically distinct populations is urgent for supplementing rye's heterotic pool. Considering that rye cultivars are panmictic populations, characterized by high levels of heterozygosity and heterogeneity, they usually display similar genetic diversity levels as landraces, namely *ex situ* collections (Persson and von Bothmer, 2000, 2002; Persson et al., 2001; Parat et al., 2016). However, conflicting results reported higher levels of genetic diversity on Portuguese rye landraces than varieties (Ribeiro et al., 2012), as expected for most crops. Previous studies on the genetic diversity of rye accessions share common features, namely: lack of correlation between accessions and geographic origin and similar genetic diversity between landraces (i.e., *ex situ*) and cultivars, which is indicative of a common genetic background, regardless of breeding level or geographical origin (Bolibok-Brągoszewska et al., 2014; Hagenblad et al., 2016; Parat et al., 2016; Targońska et al., 2016). Indeed, it was proposed that ecological and temporal isolation are key for shaping rye's genetic diversity rather than spatial or geographic isolation (Ma et al., 2004). A recent study has shown that, rather than distinction between landraces and cultivars, diversity patterns on rye seem to be related to the end use over time (Parat et al., 2016), uncovering a clear separation of rye for forage in the Mediterranean area and for grain in Northeast Europe.

SSRs have proven to be a marker of excellence for examining rye's genetic diversity (Shang et al., 2006; Akhavan et al., 2010; Gailīte et al., 2013; Parat et al., 2016; Targońska et al., 2016). Several studies using a wide range of molecular markers systems, have contributed to a better knowledge on rye's genetic diversity (e.g., Persson and von Bothmer, 2000, 2002; Chikmawati et al., 2005, 2012; Bolibok-Brbągoszewska et al., 2012; Ribeiro et al., 2012; Al-Beyroutiová et al., 2016; Parat et al., 2016; Santos et al., 2016; Targońska et al., 2016), with studies using rye cultivars and landraces, particularly from *ex situ* collections (e.g., Matos et al., 2001; Hagenblad et al., 2016; Parat et al., 2016; Targońska et al., 2016). As genetic diversity of *in situ* populations is dynamically maintained and it evolves along changing environments, evaluation of rye accessions will open insights into a new potential genetic diversity linked to the genetic adjustment on many traits during adaptation to local conditions and agriculture practices.

Our study aims at performing the first comprehensive assessment of the genetic diversity and population structuring on rye using cultivars, *ex situ* and *in situ* collections following a worldwide sampling scheme, by performing a molecular-directed analysis using SSRs markers. Data obtained exposes and delivers novel *in situ* genetic resources with potential for broadening the genetic diversity within the rye heterotic pool, thus opening a new venue for rye breeders.

## Materials and methods

### Plant material

**Figure 1:**
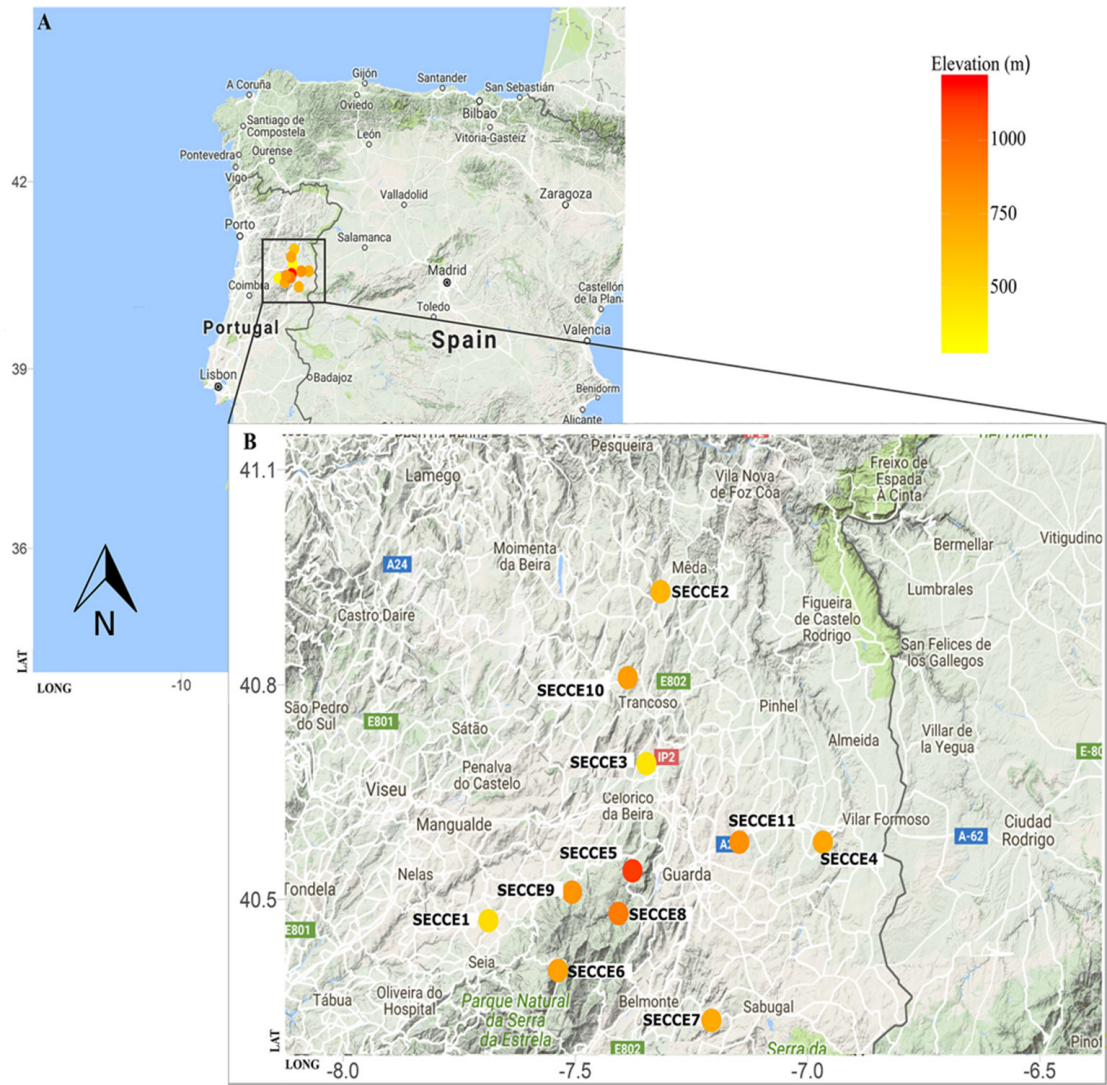
**Map of Portugal with region of rye sampling highlighted (A) and ***in situ*** populations detailed along an elevation gradient (B)**. Visualization was generated using R package *ggmap* and occurrence list according to elevation by *rgbif* package.

For this study, 28 *Secale cereale* L. subps. *cereale* accessions and its crop wild relative (*S. strictum* subps. *strictum*, referred as *S. strictum* hereforth) were selected from different geographic regions. The panel consist of eight *ex situ* accessions from gene banks, nine cultivars (“Imperial,” “Kungs II,” “Petkus,” “Dankowskie Zlote,” “Ailé,” “Voima,” “Alvão,” “Pulawskie,” and “Antoninskie”), along with 11 *in situ* accessions collected “on farm” in 2014 from Northeast Portugal (Table 1). *Ex situ* accessions were provided by the following gene banks with acronym, accession prefix and country: Leibniz-Institut für Pflanzengenetik und Kulturpflanzenforschung (IPK, R, Germany) and Nordic Genetic Resource Center (NordGen, NGB, Sweden). Rye accessions were grouped and mentioned hereforth as cultivars, *ex situ*, and *in situ/on farm* accessions. Cultivars are those resulting from modern rye breeding, *in situ* are collections held in farmers' fields and *ex situ* are those collections detained in gene banks or botanical gardens. Both *ex situ* and *in situ* accessions collectively will be referred as landraces. The 11 *in situ* Portuguese accessions were collected from local farms on Northern region (Figure 1A) where a great importance of rye culture still exists at regional level. The region sampled covers part of the Serra da Estrela Natural Park, the largest natural conservation area, and biggest mountain range in Portugal, with several valleys along the mountain assortment. Such region is characterized by harsh winters and samples selected are within 400–1100 m elevation (Figure 1B). Each accession represents a mixed sampling from small plots (maximum 0.5 ha) maintained by local farmers under a subsistence regime. The samples collected were sowed in September/October 2013 and harvested in mid-July 2014, as it is commonly performed. About 200 kg from each *in situ* population were obtained directly from each farmer and further used on genetic diversity studies and stored at the Instituto Superior de Agronomia, University of Lisbon for future characterizations of agronomical and morphological traits. All *in situ* accessions included in this study are available upon author request.

**Table 1:** **Rye sampled accessions**.

**Accession number**	**Taxon**	**Accession name**	**Location/country**	**Breeding level**	**Latitude**	**Longitude**	**Seed bank**	***N***
R2119	*Secale cereale* subsp. *cereale*	Ailé	Spain	Cultivar	38.95	−5.13	IPK	6
R 2204	*S. cereale* subsp. *cereale*	Antoninskie	Poland	Cultivar	54.37	18.64	IPK	6
R 1633	*S. cereale* subsp. *cereale*	Dankowskie Zlote	Poland	Cultivar	54.37	18.64	IPK	6
R1150	*S. cereale* subsp. *cereale*	Imperial	Canada	Cultivar	47.51	−72.11	IPK	6
R 1265	*S. cereale* subsp. *cereale*	Kungs II	Sweden	Cultivar	62	15	IPK	6
R 1667	*S. cereale* subsp. *cereale*	Petkus	German Democratic Republic	Cultivar	50.87	12.08	IPK	6
R1454	*S. cereale* subsp. *cereale*	Voima	Finland	Cultivar	64	26	IPK	6
R 751	*S. cereale* subsp. *cereale*	Pulawskie	Bazanowka/Poland	Cultivar	49.60	22.05	IPK	6
70c	*S. cereale* subsp. *cereale*	Alvão	Portugal	Cultivar	40.28	−7.74	UTAD	6
NGB14283	*S. cereale* subsp. *cereale*	Svedjeråg 66-S (Sved)	Sweden	*ex situ* accession	59	13	NORDGEN	8
18/2015	*S. cereale* subsp. *cereale*	Riodeva	Spain	*ex situ* accession	38.95	−5.13	ISA/UL	6
R 2136	*S. cereale* subsp. *cereale*	R2136Russ	Leningrad/Russia	*ex situ* accession	59.95	30.32	IPK	6
R 780	*S. cereale* subsp. *cereale*	R780Spain	Badajoz/Spain	*ex situ* accession	38.95	−5.13	IPK	6
R 2694	*S. cereale* subsp. *cereale*	R2694West	Westfalen/Germany	*ex situ* accession	52	8	IPK	6
R 1148	*S. cereale* subsp. *cereale*	R1148Turkey	Van/Turkey	*ex situ* accession	38.48	43.68	IPK	6
R 1138	*S. cereale* subsp. *cereale*	R1138Italy	Bruzolo/Italy	*ex situ* accession	45.13	7.20	IPK	6
R 1133	*S. cereale* subsp. *cereale*	R1133PT	Trás-os-Montes (PT)	*ex situ* accession	41.70	−7.13	IPK	6
02/2015	*S. cereale* subsp. *cereale*	SECCE1	Seia (PT)	*in situ* accession	40.47	−7.69	ISA/UL	16
03/2015	*S. cereale* subsp. *cereale*	SECCE2	Aveloso (PT)	*in situ* accession	40.93	−7.32	ISA/UL	16
04/2015	*S. cereale* subsp. *cereale*	SECCE3	Celorico da Beira (PT)	*in situ* accession	40.69	−7.35	ISA/UL	16
05/2015	*S. cereale* subsp. *cereale*	SECCE4	Mesquitela (PT)	*in situ* accession	40.58	−6.97	ISA/UL	16
06/2015	*S. cereale* subsp. *cereale*	SECCE5	Videmonte (PT)	*in situ* accession	40.54	−7.38	ISA/UL	16
07/2015	*S. cereale* subsp. *cereale*	SECCE6	Manteigas (PT)	*in situ* accession	40.40	−7.54	ISA/UL	16
08/2015	*S. cereale* subsp. *cereale*	SECCE7	Trancoso (PT)	*in situ* accession	40.81	−7.39	ISA/UL	16
09/2015	*S. cereale* subsp. *cereale*	SECCE8	Guarda António (PT)	*in situ* accession	40.48	−7.41	ISA/UL	16
10/2015	*S. cereale* subsp. *cereale*	SECCE9	Gouveia (PT)	*in situ* accession	40.51	−7.51	ISA/UL	16
11/2015	*S. cereale* subsp. *cereale*	SECCE10	Sabugal (PT)	*in situ* accession	40.33	−7.21	ISA/UL	16
12/2015	*S. cereale* subsp. *cereale*	SECCE11	Guarda (PT)	*in situ* accession	40.58	−7.15	ISA/UL	16
XX-0-MG-19-44650	*S. strictum (=S. montanum)*	*S. strictum*	Unknown origin^*^	Crop wild relative	–	–	BGJG/UM	5

*Accession numbers and names are provided, along taxonomic classification, breeding level, number of individuals screened (N), geographical provenance, and the seedbank from which seeds were requested (for cultivars and ex situ) or stored (in situ). IPK, Leibniz, Institut für Pflanzengenetik und Kulturpflanzenforschung (Germany); NGB, Nordic Genetic Resource Center (NordGen, Sweden), UTAD, Universidade de Trás-os-Montes e Alto Douro; BGJG/UM, Botanic Garden Johannes Gutenberg-Universität Mainz; ISA/UL, Instituto Superior de Agronomia, Universidade de Lisboa; PT, Portugal*.

* *Distribution; Mediterranean, West Asia*.

### DNA extraction

DNA from 5 to 10 individuals of each rye germplasm accessions (cultivars and *ex situ*), while for Portuguese *in situ* collections 16 individuals were used, for accounting within-population diversity. Genomic DNA (gDNA) was extracted from young leaves using the cetyltrimethylammonium bromide (CTAB) procedure adapted from Thomas et al. (1993). Briefly, young leaves (100 mg) from individual plants grown from seeds were directly ground by an Eppendorf-pestle in a 1.5 mL tube, thawed and resuspended in 300 μl of extraction buffer [0.35 M Sorbitol, 0.1 M Tris pH 8.0 NaCl, 5 mM EDTA, 1 % (w/v) PVP-40] by adding 3.8 g/L of sodium bisulphite and 1 % (v/v) 2-mercaptoethanol (Sigma Aldrich) upon use. After a 5-min incubation on ice, 300 μl of lysis buffer [0.2 M Tris pH 8.0, 2 M NaCl, 50 mM EDTA, 2% CTAB] was added along with 120 μl of 5% sarkozyl (w/v) and RNAse A (10 mg/mL). After incubation at 65°C for 15 min, an equal volume of chloroform: isoamyl alcohol (24:1) was then mixed by a brief vortex, and the aqueous phase was recovered by centrifugation at 16,000 g for 15 min. gDNA was precipitated with 0.6 volume of isopropanol and recovered by a 15-min centrifugation at 16,000 g, followed by a washing step with 70% ethanol. Following a centrifugation at 16,000 g for 10 min, the pellet was dried and resuspended in 40 μl deionized water. DNA purity and concentration were measured at 260/280 nm using a spectrophotometer (NanoDrop-1000, Thermo Scientific) while DNA integrity was verified by agarose gel electrophoresis.

### Microsatellite genotyping

A set of 16 microsatellite markers (Supplementary Table S1) was used for screening rye accessions, consisting of nine genomic SSRs (gSSRs, Saal and Wricke, 1999) and seven Expressed Sequence Tags-SSRs (ESTs-SSRs, Hackauf and Wehling, 2002). Before multiplexing, each SSR marker was validated in singleplex polymerase chain reactions (PCR) using a three-primer PCR approach (*sensu* Schuelke, 2000) for reaction reproducibility and presence of PCR artifacts. Each SSR was PCR amplified in a 25 μl volume reaction following cycling conditions previously described (Saal and Wricke, 1999; Hackauf and Wehling, 2002), using HotStarTaq DNA Polymerase kit (Qiagen), as per manufacturer's instructions. After, SSRs amplified fragments were run in an ABI 3130XL sequencer (Applied Biosystems) with the internal size standard GS500 LIZ (Applied Biosystems), while allele calling was performed in GeneMapper v 3.7 (Applied Biosystems). Stringent selection of markers to ensure the success of co-amplification loci using Multiplex Manager software (Holleley and Geerts, 2009), allowed building four SSRs panels assembled in 4-plex PCR reactions (Multiplex A, B, C, and D; Table 2), using four universal forward fluorescently labeled primers following Culley et al. (2013). To increase genotyping accuracy, a “Pig-tail” sequence was added at the 5′ end of each of the reverse primer (Brownstein et al., 1996). PCR multiplex amplifications were carried out using the QIAGEN Multiplex PCR kit (Qiagen), following the manufacturer' s protocol, in a total volume of 25 μL with 1 μL of gDNA (50–100 ηg) and 2.5 ρmol of each primer Forward and Reverse and 0.15 ρmol of each of the tailed fluorescently labeled primers (D1–D4). Reactions were done in 96 well-plates and on each plate one sample was repeated per run thus working as positive control for scoring. Negative PCR controls were included. Initially, a hot-start step at 95°C for 15 min was performed, followed by a touchdown cycling protocol adapted from Hackauf and Wehling (2002): 5 cycles of denaturation at 95°C for 45 s, primer annealing at 68°C for 5 min with −2°C/cycle; a sequence extension at 72°C for 1 min; 5 cycles of denaturation at 95°C for 45 s, primer annealing at 58°C for 2 min with −2°C/cycle and an extension step for 1 min at 72°C; 27 cycles at 95°C for 45 s, 47°C for 75 s, and 72°C for 1 min; followed by a final extension step at 72°C for 10 min. Multiplex PCR products were run as described earlier and SSR allele sizes were aligned with the internal size standard and scored using the binning function in GeneMapper v3.7. (Applied Biosystems). To improve the SSR marker data quality, allele assignments were checked manually, and ambiguous results were set as “missing data.”

**Table 2:** **Markers diversity measurements**.

**Locus**	**Sample analyzed**	**Allele number**	**PIC**	**He**	**H_O_**	***F***	**Frequency null alleles (>0.20)**
**ESTs-SSRs (Hackauf and Wehling**, **2002****)**							
SCM113	284	1	–	–	–	–	–
SCM166	285	4	0.45	0.48	0.51	−0.28	–
SCM63	255	12	0.73	0.76	0.54	0.02	0.26 SECCE10, 0.21 SECCE2, 0.25 SECCE8
SCM152	285	11	0.83	0.85	0.57	0.06	0.25 Dankow
SCM98	285	3	0.47	0.55	0.61	−0.41	–
SCM164	268	11	0.67	0.70	0.68	−0.16	0.25 R1138Italy
SCM66	285	3	0.39	0.51	0.80	−0.73	–
Mean		7.33	0.59	0.64	0.62	−0.25	–
**gSSRs (Saal and Wricke**, **1999****)**							
SCM39	281	10	0.63	0.66	0.54	0.06	0.21 Alvão, 0.25 R1138Italy, 0.25 R1148Turkey
SCM2	284	5	0.52	0.58	0.62	−0.34	–
SCM28	278	13	0.81	0.83	0.71	−0.05	–
SCM9	282	7	0.65	0.70	0.74	−0.25	–
SCM75	261	11	0.82	0.84	0.78	−0.15	0.22 R1148Turk
SCM43	284	9	0.79	0.81	0.79	−0.11	–
SCM138	285	10	0.85	0.86	0.80	−0.12	0.21 R1133PT
SCM86	285	12	0.77	0.80	0.85	−0.28	–
Mean		9.6	0.73	0.76	0.73	−0.15	–
Total Mean		8.64	0.67	0.71	0.68	−0.20	
Total		122					

*The level of genetic diversity of each SSR marker was described with the parameters number of alleles, Polymorphism Information Content (PIC), gene diversity (expected heterozygosity, He), observed heterozygosity (H_O_), inbreeding/fixation coefficient (F), and frequency of null alleles above 0.20 as calculated by FreeNa are presented with additional information on the identified population*.

### Genetic diversity—based analysis

Genotyping errors were assessed using MICRO-CHECKER v2.2.3. (Van Oosterhout et al., 2004), and estimation of null alleles frequency was done with the EM algorithm of Dempster et al. (1977) as implemented in FreeNA (http://www.montpellier.inra.fr/URLB/). These values were computed, as described in Chapuis and Estoup (2007), with 10,000 bootstrap iterations, alternatively using and not using the Excluding Null Alleles (ENA) method, after assessment of null allele frequencies. Polymorphism information content (PIC) and genetic diversity indices were calculated with Microsatellite Toolkit v.3.1.1 (Park, 2001) and GenALEx 6.5 (Peakall and Smouse, 2006, 2012). These included the total allele number and mean alleles per locus (*N*_*a*_), private alleles, inbreeding coefficient (fixation index, *F*), observed (*H*_*O*_), and expected (*H*_*E*_) heterozygosity. Deviations from Hardy–Weinberg equilibrium (HWE) were assessed for each locus-population combination and linkage disequilibrium (LD) to determine the extent of distortion from independent segregation of loci using GenePop v4.5 (Rousset, 2008). Statistical significance for both HWE and LD was tested by running a Monte Carlo Markov Chain (MCMC) consisting of 10,000 iterations each, and *p*-values were corrected for multiple comparisons [*p* < 0.00012, (0.05/406)] by applying a sequential Bonferroni correction (Rice, 1989).

To detect isolation-by-distance (IBD) effects, *F*_ST_/(1 – *F*_ST_) and $F_{\text{ST}}{}^{\text{ENA}}$/(1 – $F_{\text{ST}}{}^{\text{ENA}}$) matrixes were done, with a geographic distance matrix defined as pairwise distances generated from geographical coordinates expressed in Km. Pairwise unbiased *F*_ST_-values using the ENA method ($F_{\text{ST}}{}^{\text{ENA}}$) for each population comparison were calculated with FreeNA software, while *F*_ST_- values were generated in GenALEx 6.5. Both Slatkin's linearized *F*_ST_ (*F*_ST_/(1 − *F*_ST_)) matrixes were obtained in GenoDive 2.0b27 (Meirmans and Van Tienderen, 2004). The correlation between the two data matrices was assessed using a Mantel test and its significance estimated by *p*-values, the regression coefficient (*R*^2^), and the mean correlation coefficient (*R*_XY_) over 999 random permutations as implemented in GenALEx 6.5.

### Population structure

Population structure was addressed using three approaches: (i) estimating relations among populations using genetic distances; (ii) hierarchical genetic analysis by AMOVA; and (iii) individual-based clustering.

#### (i) Estimating relations among populations using genetic distances

Relationships among populations were estimated with Cavalli-Sforza and Edward's chord genetic distances (*DC*, Cavalli-Sforza and Edwards, 1967) using the INA method computed in FreeNA (*DC*^INA^), and Nei's distance (*D*, Nei, 1972) calculated in GenALEx 6.5. Unweighted Pair Group Method with Arithmetic Mean (UPGMA) and Neighbor-Joining (NJ) trees were produced using package *ape* v3.4. (Paradis et al., 2004) for R v3.2.3 (R Developmental Core Team, 2015) based on 10,000 bootstraps values assessed by *aboot* function from *poppr* v2.1.0. package (Kamvar et al., 2014, 2015). Trees were further edited in FigTree v1.4.2 (Rambaut, 2014).

#### (ii) Hierarchical genetic analysis (AMOVA)

The hierarchical distribution of genetic variation on the 28 populations (excluding the outgroup *Secale strictum*) was characterized using Analysis of Molecular Variance (AMOVA, Weir and Cockerham, 1984; Excoffier et al., 1992; Hill, 1996) with ARLEQUIN v3.5.1.3 (Excoffier and Lischer, 2010) and significance was assessed after 1000 permutations. Two 3-level AMOVAs were pursued: one using cultivars and landraces (i.e., *ex situ* and *in situ* accessions) as groups, and the second, narrowed to landraces using *ex situ* and *in situ* accessions alone. In each AMOVA, the total variance was partitioned into components to account for differences between two defined groups [*V*_a_, (1) cultivars and landraces; (2) *ex situ* vs. *in situ* accessions], differences among populations within those groups (*V*_b_), differences among individuals within populations (*V*_c_). Variance components (*V*_a_, *V*_b_, and *V*_c_) were used to calculate the fixation indices (*F*-statistics; *F*_CT_, *F*_SC_, *F*_ST_) according to Weir and Cockerham (1984).

#### (iii) Individual-based clustering

To identify genetically distinct clusters, two individual-based assignment approaches were pursued: a bayesian clustering analysis using STRUCTURE (Pritchard et al., 2000) and a multivariate analysis by Discriminant Analysis of Principal Components (DAPC, Jombart et al., 2010). While STRUCTURE uses allele frequency and LD information from the dataset directly; the latter is a multivariate method which attempts to summarize the genetic differentiation between groups, while overlooking within-group variation and not relying on a particular population genetics model and free of HWE assumptions (Jombart et al., 2010).

Bayesian model-based clustering algorithm implemented in STRUCTURE v.2.3.4 was used to identify genetic clusters under a model assuming admixture and correlated allele frequencies without using population information. An exploratory run was performed setting *K*-values from 1 to 30 with a 50,000 burn-in period followed by 100,000 MCMC iterations. Subsequent runs were set for a burn-in period length to 100,000 followed by 1,000,000 MCMC iterations with *K*-values narrowed from 1 to 10 with 10 runs computed for each *K*. StructureHarvester v0.6.94 (Earl and VonHoldt, 2012) was then used to calculate Δ*K ad hoc* statistics from Evanno et al. (2005) for estimating the most likely *K*-value, which is based on the rate of change of the “estimated likelihood” between successive *K*-values. CLUMPP v1.1.2 (Jakobsson and Rosenberg, 2007) was used to average replicate runs for the selected *K*-value, for accounting problems with multimodality and label switching between iterations of STRUCTURE runs. CLUMPP results were then plotted with DISTRUCT v1.1 (Rosenberg, 2004).

DAPC was implemented in R using *adegenet* v1.3.1 package (Jombart, 2008). The function *find.clusters* was used to find the ideal *K*-value, based on the computation of Bayesian Information Criterion (BIC) scores, maintaining default parameters and retaining all principal components (PCs). Cross validation using the *xvalDapc* function was pursued to determine the optimal number of PCs to retain in the Discriminant Analysis (DA).

## Results

### SSRs genotyping and statistics

All 16 SSRs were tested in singleplex reactions at the estimated optimal annealing temperature, and only after this initial quality assessment, SSRs markers were grouped into 4-plex reactions (Supplementary Table S1). Upon multiplex reactions, SCM180 (Multiplex C) displayed a difficult allele scoring performance, not depicted in singleplex reactions. This fact may be ascribed to PCR dynamics under a multiplex reaction, where concentrations of different primers are equimolar, requiring in some cases, relative balanced primers concentrations (Sint et al., 2012). For the remaining 15 SSRs loci, allele profiles were clear and easy to score. No errors in the genotypic data matrix were detected, indicating the absence of potential errors associated with stuttering bands or large allele dropout in SSRs screened. In only 10 of the 406 locus-populations comparisons, the frequencies of null alleles were higher than 0.20 (Table 2). Deviations from Hardy–Weinberg Equilibrium (HWE) were observed in most loci except for SCM166, with 70 locus-population combinations statistically significance (*p* < 0.05); while after sequential Bonferroni correction only four loci (SCM64, SCM39, SCM63, SCM75) displayed significant deviations, matching 11 of the 406 locus-population combinations (Supplementary Table S2). All 15 loci were in linkage equilibrium after Bonferroni correction, thus being non-correlated, and alleles independently segregated and inherited (data not shown). Negative fixation index (*F*) estimates were observed across several loci, exceptions for the EST-SSRs SCM152 (0.060), and SCM63 (0.017) and in the gSSR SCM39 (0.057, Table 2), which can reflect more heterozygotes than expected or other population structure complexities.

### Genetic diversity estimates

Overall, a total of 122 alleles were detected in the 285 individuals analyzed (Table 2). All loci screened were polymorphic except SCM113, which revealed to be monomorphic (194 bp, Supplementary Table S1) being not used for further analysis. The total number alleles per locus ranged from 3 (SCM66 and SCM98) to 13 (SCM28) with an average of 8 alleles per locus (Table 2). When comparing gSSRs with EST-SSRs, the first revealed a higher number (5–13) of alleles per locus than EST-SSRs (3–12), with an average of 9.6 and 7.3 alleles, respectively (Table 2). Once the dataset was separated in cultivars and landraces (including *in situ* and *ex situ* accessions) accessions, results showed that in landraces the mean allele number is higher with 4.4 in all SSRs, 4.8 with gSSRs, and 4.4 in EST-SSRs, against the values in cultivars (3.3 alleles per locus in all SSRs, 3.7 in gSSRs, and 3.3 for EST-SSRs, Table 3). When analyzing landrace dataset, *ex situ* collections (2.97 all SSRs, 3.3 for gSSRs, 2.97 for EST-SSRs, Table 3) showed a substantially lower mean of alleles per locus when compared to *in situ* accessions (5.5 all SSRs, 6 for gSSRs, 5.5 for EST-SSRs), but similar values to those obtained for cultivars.

**Table 3:** **Genetic diversity analysis by cultivars and landraces (i.e., ***ex situ*** and ***in situ***)**.

	***N***	**Loci**	**He**	**He SD**	**H_O_**	**H_O_ SD**	***N_a_***	***N_a_* SD**	***F***
**ALL SSRs**									
Cultivars	54	14	0.61	0.04	0.70	0.05	3.28	1.20	−0.26
*ex situ*	50		0.56	0.05	0.67	0.05	2.97	1.06	−0.33
*in situ*	176		0.67	0.04	0.68	0.03	5.47	2.20	−0.07
Landraces	226		0.63	0.05	0.67	0.04	4.42	1.72	−0.18
Total	285		0.61	0.05	0.68	0.04	3.98	1.53	−0.23
**gSSRs**									
Cultivars	54	8	0.68	0.04	0.73	0.06	3.8	1.17	−0.18
*ex situ*	50		0.62	0.06	0.69	0.06	3.3	1.05	−0.24
*in situ*	176		0.70	0.04	0.74	0.04	6.0	1.95	−0.10
Landraces	226		0.67	0.05	0.72	0.05	4.8	1.57	−0.16
Total	285		0.67	0.05	0.72	0.05	4.4	1.44	−0.19
**EST-SSRs**									
Cultivars	54	6	0.61	0.04	0.70	0.05	3.28	1.20	−0.14
*ex situ*	50		0.56	0.05	0.67	0.05	2.97	1.06	−0.20
*in situ*	176		0.67	0.04	0.68	0.03	5.47	2.20	−0.12
Landraces	226		0.63	0.05	0.67	0.04	4.42	1.72	−0.16
Total	285		0.60	0.04	0.67	0.04	3.91	1.50	−0.16

*Data are provided by total SSRs, gSSR, and EST-SSRs, following by the grouping scheme adopted, with sample size (N): cultivars, ex situ, and in situ collections, which collectively are referred as landraces and total sampling (i.e., sampled accessions including S. strictum). Genetic diversity indices for each group was assessed by expected heterozygosity (He) and observed heterozygosity (H_O_) with corresponding standard deviation (SD) values, inbreeding/fixation coefficient (F), and mean alleles per locus (N_a_)*.

Overall, Polymorphic Information Content (PIC) values ranged from 0.39 (SCM66) to 0.85 (SCM138) with a mean value of 0.67 (Table 2). Average PIC-values were higher in gSSRs (PIC_gSSRs_ = 0.73) than in EST-SSRs (PIC_EST-SSRs_ = 0.59). Similar PIC-values were observed in cultivars, ranging from 0.366 (SCM63) to 0.695 (SCM138), and in landrace accessions (SCM166: PIC = 0.346; SCM138: PIC = 0.677). In both cultivars and landraces, gSSRs revealed to be more polymorphic than EST-SSRs. In our 14-loci dataset, observed heterozygosity (H_O_) varied from 0.51 (SCM166) to 0.85 (SCM86) with a mean of 0.68 (Table 2); and expected heterozygosity (He) varied between 0.480 (SCM166) and 0.86 (SCM138). When narrowing analysis to gSSRs and EST-SSRs, both mean H_O_ and He were higher in genomic (H_O_ = 0.73; He = 0.76) compared to the expressed loci (H_O_ = 0.62; He = 0.64).

The Fixation Index *F* (also called the Inbreeding Coefficient) exhibits values from −1 to +1. Values close to zero are expected under random mating, while substantial positive values indicate inbreeding or undetected null alleles. Negative values denote excess of heterozygosity, due to negative assortative mating, or selection for heterozygotes. Overall, negative *F*-values were observed across most accessions (Table 3), thus revealing an outbreeding scenario with increased number of heterozygotes. Positive *F* was encountered in six Portuguese *in situ* accessions (SECCE1–SECCE6), ranging from 0.002 (SECCE5) to 0.049 (SECCE1; Supplementary Table S3). Despite positive, their relatively small *F*-values indicate that these populations are at or near Hardy–Weinberg equilibrium, further supported by the lower observed heterozygosity values against the expected under HWE (Supplementary Table S3).

Pairwise Wright's *F*-statistics (*F*_ST_) was used as a measure of the extent of genetic differentiation among subpopulations, with values ranging from 0 (no differentiation) to 1 (high differentiation). Mean pairwise *F*_ST_ was 0.136 (min = 0.017, “Pulawskie” and “Dankowskie”; max = 0.418, “Riodeva” and *S. strictum*) indicating an overall low level of population's differentiation (Supplementary Table S4). Most pairwise populations (749 out of 784) showed low to moderate genetic differentiation (pairwise *F*_ST_ < 0.250), while the remaining 35 pairwise populations displayed high genetic differentiation (pairwise *F*_ST_ ranged from 0.251 to 0.418). The presence of null alleles has not caused a significant overestimation of the level of population differentiation, as low population differentiation was also depicted with $F_{\text{ST}}{}^{\text{ENA}}$ (mean = 0.138; min = 0.015, “Pulawskie” and “Dankowskie”; max = 0.417, “Riodeva” and *S. strictum*), with 747 pairwise populations showing low to moderate genetic differentiation (Supplementary Table S4). The remaining 37 pairwise comparisons exhibited high genetic differentiation (pairwise $F_{\text{ST}}{}^{\text{ENA}}$ > 0.250). Overall, cultivars (mean *F*_ST_ = 0.136; $F_{\text{ST}}{}^{\text{ENA}}=0.138$) and *ex situ* collections (mean *F*_ST_ = 0.163; $F_{\text{ST}}{}^{\text{ENA}}=0.165$) presented a moderate population differentiation, whereas *in situ* accessions almost no genetic differentiation were depicted (mean *F*_ST_ = 0.057; $F_{\text{ST}}{}^{\text{ENA}}=0.059$). An overestimation of *F*_ST_-values due to null alleles is widely known especially in cases of significant population differentiation, which is not the case in our study where weak population differentiation was detected.

**Figure 2:**
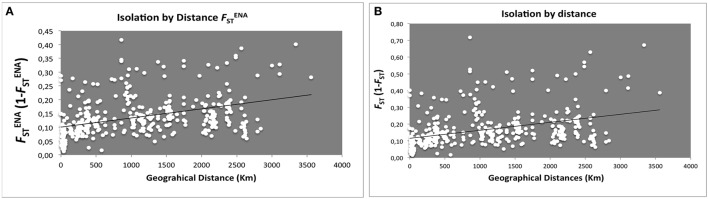
**Scatter plots of genetic distance vs. geographical distance for pairwise population comparisons**. Each point represents one population pairwise using Slatink's linearized **(A)** $F_{\text{ST}}{}^{\text{ENA}}$/(1 − $F_{\text{ST}}{}^{\text{ENA}}$; *R*^2^ = 0.1612, *R*xy = 0.402, *P* = 0.001; 999 permutations) and **(B)** *F*_ST_/(1 − *F*_ST_; *R*^2^ = 0.14067, *R*xy = 0.375, *P* = 0.002; 999 permutations) plotted against geographic distance (Km).

In order to understand whether genetic variation is correlated with geographical gradients, Isolation-by-distance (IBD) effects were addressed. *F*_ST_-values (Figure 2A either excluding, $F_{\text{ST}}{}^{\text{ENA}}$, or not null alleles *F*_ST_ Figure 2B) confirmed a small, yet significant, explanation of genetic diversity variation across a geographic range.

### Population structure

#### (i) Estimating relations among populations using genetic distances

**Figure 3:**
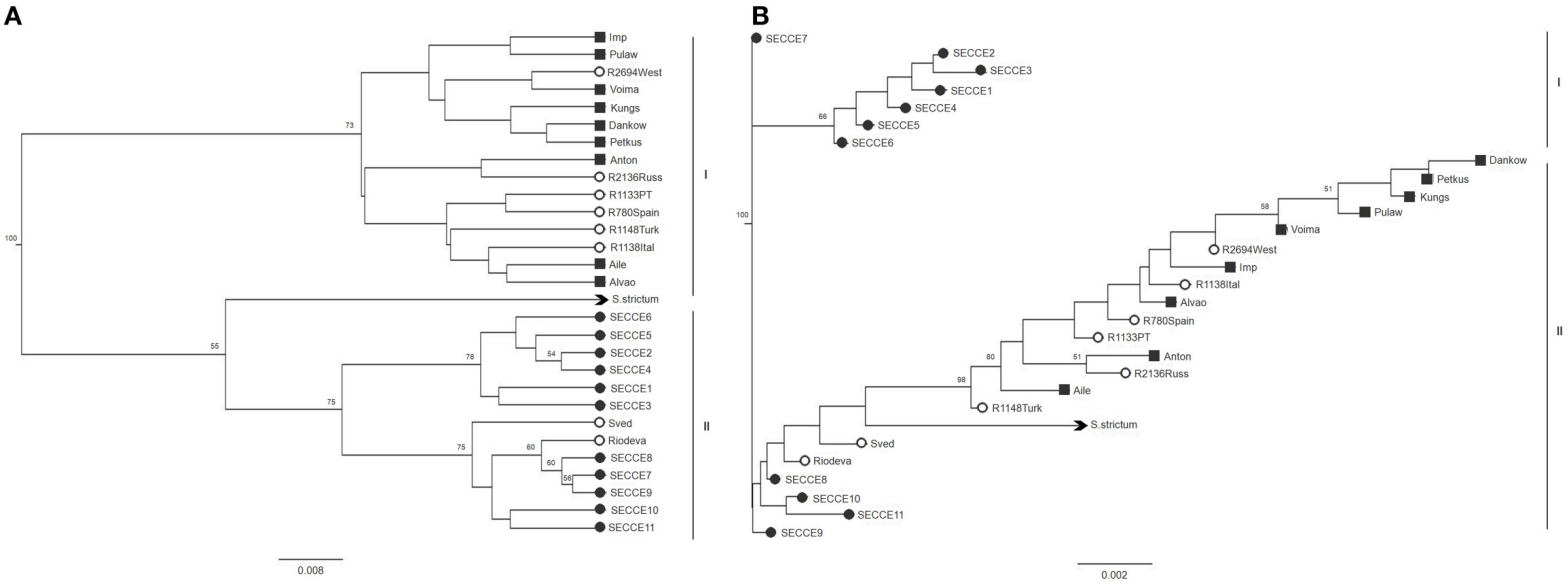
**UPGMA (A) and NJ (B) trees generated from Nei's ***D*** distance matrix**. Accessions are indicated by symbols reflecting grouping assignment: ■ cultivars, 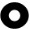 *ex situ* accessions, 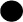 *in situ* accessions. *S. strictum* was used as a species outgroup (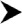). Only bootstraps values above 50 are indicated.

UPGMA and NJ trees were built using Nei's *D* and *DC*^INA^ genetic distances (Supplementary Table S5) across accessions screened. Regarding UPGMA trees, similar structure was observed with both *D* and *DC*^INA^ matrices, thus indicating a reliable topology regardless of the different genetic distances algorithms used. As such, only Nei's *D* distances matrices—derived trees are presented in Figure 3. In UPGMA-derived tree, two clusters are depicted (Figure 3A): one comprising all cultivars with most of *ex situ* accessions and another clade comprising *in situ* accessions, “Sved” and “Riodeva” *ex situ* samples and *S. strictum*. No clade seems to cluster accessions on the basis of a particular geographic origin. NJ dendogram derived from Nei's distance matrix, grouped Portuguese landraces into different clusters (Figure 3B), displaying a different population structuring of *in situ* accessions compared to UPGMA trees: one group (i.e., SECCE1–SECCE5) without any link with other rye accessions and the other Portuguese accessions placed within the same clade as cultivars and *ex situ* accessions.

Conversely to UPGMA-derived dendograms, the two genetic distance algorithms produced very dissimilar NJ-generated trees (Nei's *D* distance, Figure 3; *DC*^INA^ Supplementary Figure S1), which may be attributed to different assumptions adopted in each clustering methods, with a strict (UPGMA) or relaxed (NJ) molecular clock shown previously to have implications when inferring phylogenies considering that rates of evolution may vary among microsatellite loci (Putman and Carbone, 2014).

#### (ii) Analysis of molecular variance

When grouping cultivars vs. landraces (*ex situ* and *in situ* accessions), AMOVA results showed that molecular variation was mainly (86.84%) found within accessions, whereas variation among accessions within groups explained 8.86% and variance among groups represents only 4.30% of the total genetic variability (Table 4). Regarding *ex situ* vs. *in situ* accessions, a similar scenario was depicted, with genetic variation being higher within accessions (89.64%), rather than among groups (2.86%) or within groups (7.49%). In both cases, a high molecular variation was found within accessions, as expected for a cross-pollinated species, as previously detected in other rye studies (Gailīte et al., 2013; Hagenblad et al., 2016; Parat et al., 2016; Targońska et al., 2016).

**Table 4:** **AMOVA results including fixation indices ***F***_**CT**_, ***F***_**SC**_, and ***F***_**ST**_**.

**Source of variation**	***df***	**Sum of squares**	**Variance components**	**Variation (%)**	**Fixation indices**
**CULTIVARS vs. LANDRACES**					
Among groups	1	40.572	*V*_a_ = 0.211	4.30	*F*_CT_ = 0.132^*^
Among accessions within groups	26	337.191	*V*_b_ = 0.435	8.86	*F*_SC_ = 0.093^*^
Within accessions	532	2265.938	*V*_c_ = 4.260	86.84	*F*_ST_ = 0.043^*^
***EX SITU* vs. *IN SITU* ACCESSIONS**					
Among groups	1	35.693	*V*_a_ = 0.137	2.86	*F*_CT_ = 0.029^*^
Among accessions within groups	19	235.926	*V*_b_ = 0.358	7.49	*F*_SC_ = 0.077^*^
Within accessions	455	1949.188	*V*_c_ = 4.284	89.64	*F*_ST_ = 0.104^*^

*The genetic differentiation among cultivars vs. landraces (i.e., ex situ/in situ accessions) and ex situ vs. in situ accessions groups is denoted as F_CT_, among accessions within groups as F_SC_ and within accessions as F_ST_*.

* *p < 0.001*.

#### (iii) Individual based-clustering using bayesian and a multivariate discriminant analysis to uncover population structure

**Figure 4:**
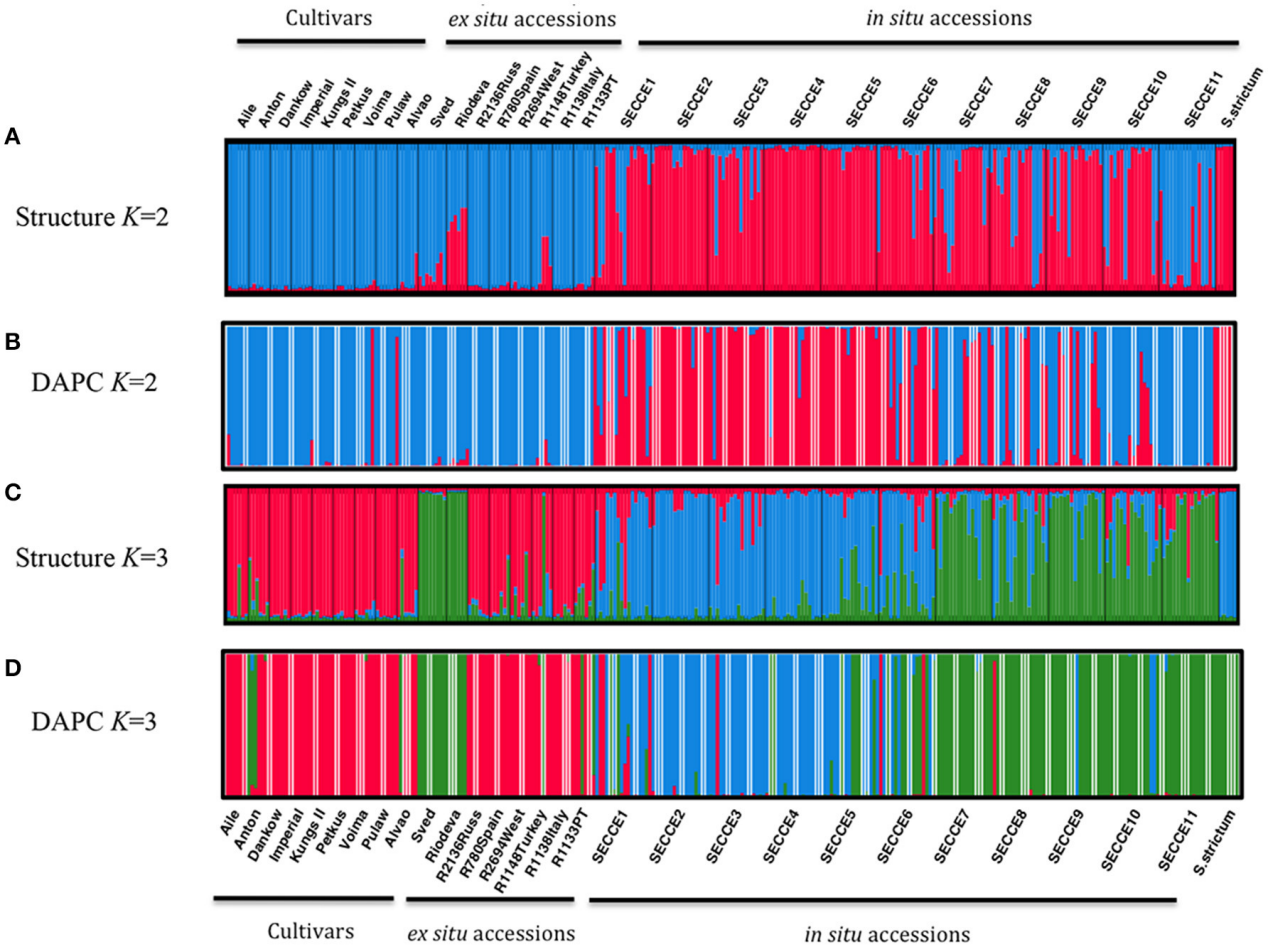
**Clustering based on SSR data using STRUCTURE (***K*** = 2, A; ***K*** = 3, C) and DAPC (***K*** = 2, B; ***K*** = 3, D) analysis**. Each rye accession is organized following the grouping as cultivars, *ex situ* and *in situ* accessions. The length of each section is proportional to the estimated ancestry value of the individual accession to each one of the *K* clusters for STRUCTURE and memberships probabilities for DAPC analysis. Each individual is represented as a vertical bar according to each *K* sections. Thin black vertical lines separate different accessions. Labels on the *x*-axis indicate rye accessions IDs.

**Figure 5:**
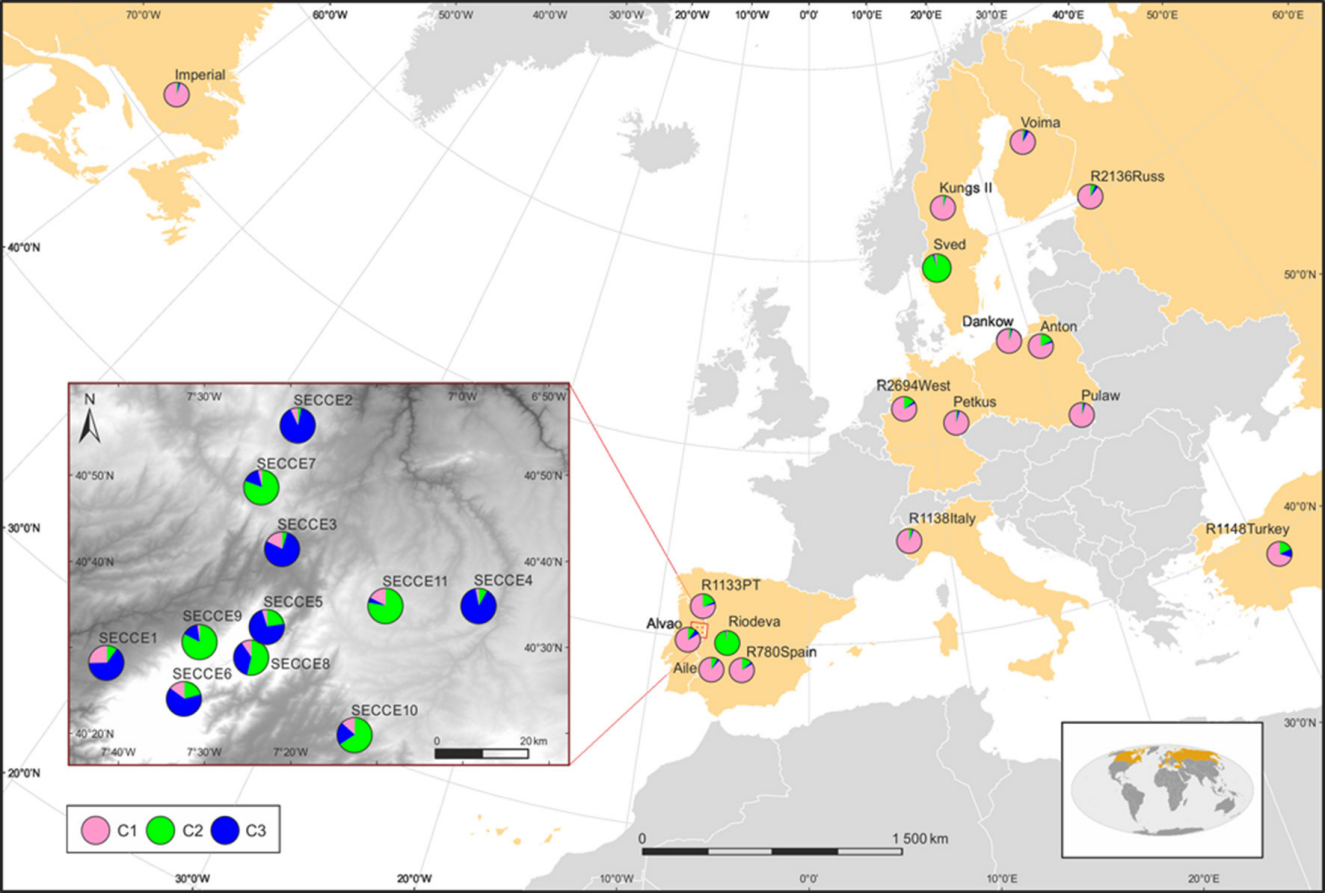
**Geographical distribution of clusters according to the ***K*** = 3 model in STRUCTURE**. Pie charts represent the sum of all individuals' membership in each cluster at each locality on the map, as identified by the probability assignment defined by STRUCTURE analysis, using ArcGIS 10.3 (ESRI). Population codes are indicated in each pie chart.

Exploratory STRUCTURE run considering the biggest range of clusters conceivable (*K* = 1–30), determined *K* = 2 as the most likely model, following Evanno et al. (2005) Δ*K* method, with no clear plateau observed in the Ln P(D) = L(*K*) for each value of *K* (data not shown). Particularly, *K*-models above 10 revealed high standard deviations of log-likelihood along low values following Δ*K ad hoc* statistics, thus showing that these cluster assignment are not reliable to describe our dataset. Though, this exploratory STRUCTURE results prompted us to subsequently constraint runs to 10 possible clusters (*K* = 1–10). This analysis assigned *K* = 2 as the optimal number of groups based on Δ*K*, with *K* = 3 also displaying high Δ*K*-values (Supplementary Figure S2). In *K* = 2, cultivars were grouped in a single cluster (blue cluster), along with the *ex situ* collections; whereas *in situ* are grouped essentially in a second cluster (pink cluster) together with *S. strictum* (Figure 4A). It is worth mentioning that the *in situ* accession SECCE11 seems to be genetically clustered within blue cluster, along with cultivars and *ex situ* accessions, with some admixed individuals. The result of *K* = 3 was also analyzed (Figure 4C) as the next most likely model. In this assignment, cultivars along most *ex situ* accessions are grouped into a single cluster (pink cluster, C1), excluding “Sved” and “Riodeva” grouped in a different cluster (green cluster, C2), as in *K* = 2, with few or even no admixture. *In situ* populations SECCE7 to SECCE11 are assembled in the green cluster (C2), together with “Sved” and “Riodeva” *ex situ* accessions, with the occurrence of other admixed *in situ* accessions (SECCE1–SECCE6), which belong to the blue cluster (C3, Figure 5). Interestingly, *S. strictum* was assigned to the blue cluster along with some Portuguese admixed accessions (SECCE1–SECCE6), thus reflecting a common genetic diversity with the rye crop wild relative that might be linked to preservation of an ancient diversity resulting from the low diversification rate of Portuguese accessions.

DAPC analysis was made without any *a priori* group assignment. To infer the appropriate number of genetic clusters, the lowest Bayesian Information Criteria (BIC) score was selected, predicting a *K* = 3 (Supplementary Figure S3). Cross validation using the *xvaldapc* function outcome the number of PCA axes retained against the proportion of successful outcome prediction, which allowed retaining 60 PCA axes (considering the highest successful assignment- 93.41%, with the lowest mean squared error, MSE- 7.76%) and 2 Discriminant Functions (explaining 92.9% of cumulative variance), for inferring the 3 genetic clusters. When displaying loading plots from both discriminant functions, one can determine which variables (i.e., alleles/loci) contributed the most for the three-clustering assembly. As such, alleles 177 (SCM138), 369 (SCM152), 245 (SCM166) from DF1, and alleles 147 and 150 from SCM164 together with 182 (SCM75), 171 (SCM138), and 194 (SCM166) from DF2 are responsible for most of the genetic variation explaining the three genetic cluster assignment (Supplementary Figure S3). A scatterplot allows an the overview of the 3 genetic groups clustering (Supplementary Figure S4), and when performing a DAPC membership probability plot as in STRUCTURE (Figure 4D), one can depict a similar clustering assignment as determined with *K* = 3 in STRUCTURE: pink cluster with cultivars and most *ex situ* populations, green cluster with SECCE7–11 *in situ* populations along *ex situ* “Riodeva” and “Sved” populations and *S. strictum*, and blue cluster comprising the remaining *in situ* populations (SECCE1–SECCE6). Analysis of *K* = 2 from DAPC (Figure 4B) was also performed in order to obtain a comparison with the ideal *K* inferred from STRUCTURE analysis. For this *K* clustering, cross-validation analysis following the Occam's razor principle determined the retention of 20 PCA axes (94.56% of successful assignment with 7.67% of MSE), capturing 60% of cumulative variance, and with only 1 Discriminant Function for describing the 2 genetic clusters (data not shown). Following this cluster assignment, two different genetic backgrounds of Portuguese *in situ* accessions can be depicted: one group (SECCE7–SECCE11) that shares allelic diversity with cultivars and *ex situ* material in the blue cluster, and other group (SECCE1–SECCE6) grouped with *S. strictum* in the pink cluster, thus showing a common genetic diversity background with wild rye (Figure 4B).

Considering the overall pattern of genetic clustering and observed intraspecific variation, STRUCTURE, and DAPC produced similar results, showing optimal clustering of individuals that separates most cultivars and *ex situ* from Portuguese *in situ* accessions.

## Discussion

In this study, 28 rye accessions screened with SSRs included nine international rye cultivars, eight worldwide *ex situ* accessions and 11 *in situ* accessions from Northeast Portugal, one of the regions of excellence for rye cultivation in this country. Also, wild rye *S. strictum* was included in order to track any shared allelic diversity existing with the screened accessions. The observed genetic diversity and population structure of a global collection of rye cultivars and *ex situ* accessions along an exhaustive sampling of *in situ* Portuguese accessions indicate that (i) rye's genetic diversity do not follow a geographical/spatial trend (ii) *in situ* accessions display similar genetic diversity than cultivars/*ex situ* collections yet with a different genetic background and (iii) exists an unexplored genetic diversity on *in situ* accessions which can represent an effective alternative to increment rye heterotic pool in future breeding programs.

### SSRs performance and informativeness

Twenty-nine rye accessions were first genotyped with 16 SSRs, and after SSRs quality assessment 14 markers were further used for subsequent genetic diversity analysis (Supplementary Table S6). One SSR was discarded due to low reproducibility in a multiplex PCR amplification (SCM180) and other revealed to be monomorphic (SCM113) across the germplasm analyzed and thus not being an informative marker for posterior diversity analysis. Unexpectedly, no private alleles were detected, contrasting with other SSRs studies in rye populations (Parat et al., 2016; Targońska et al., 2016). In this context, by not detecting private alleles, one can speculate that alleles are present in one or more rye populations regardless of being cultivars or landraces thus representing significant shared allelic diversity. Observed heterozygosity (H_O_) obtained in our study (0.51, SCM166–0.85, SCM86) with a mean of 0.68, is higher than those reported for 9 Latvian rye accessions using 9 genomic loci (Gailīte et al., 2013) with a mean H_O_ of 0.58, ranging from 0.21 (SCM2) to 0.84 (SCM9). Furthermore, a recent study using 32 SSRs in 14 rye accessions (Parat et al., 2016), reported values of H_O_ per SSR of 0.44 ± 0.17 and 0.67 ± 0.14 for He; while in our study, despite using only 14 SSRs both H_O_ and He were similar (0.64 ± 0.21 and 0.66 ± 0.22, respectively) likely due to a higher number of rye populations screened (*n* = 29).

PIC-values obtained for SSRs used were high (average PIC = 0.67) which indicates their high informativeness, which was predictable since the SSRs markers were not selected randomly, but based on the previous performance analyses (Shang et al., 2006). Our PIC-values were higher when compared with a recent study using 22 SSRs (including EST-SSRs and gSSRs) to screen 367 Polish rye accessions, which displayed a 0.57 average PIC-value for all loci used (Targońska et al., 2016); but similar to those obtained in 14 rye accessions using 32 SSRs (PIC-values ranged from 0 to 0.92) with an average of 0.62 (Parat et al., 2016). We detected higher PIC-values in gSSRs (0.73) than in EST-SSRs (0.59), which is in agreement with a previous study using 8 gSSRs (PIC = 0.63) and 14 EST-SSRs (PIC = 0.54; Targońska et al., 2016). Inversely, another analysis of rye cultivars using both gSSRs and EST-SSRs, showed a lower polymorphism content in 13 genomic SSRs (0.38) than in 11 EST-SSRs (0.58; Shang et al., 2006). Deviations on PIC-values depicted in our study might be due to differences in plant material sources compared to former reports, which may influence the number of alleles detected at each SSR locus, though a potential influence of the lower number of SSRs loci used should not be discarded. When analysing null alleles presence and their effect on population structure, only 10 of the 406 loci harbored null alleles with a frequency higher than 0.20, and as such, no overestimation of *F*_*ST*_ due to null alleles was observed (mean $F_{\text{ST}}{}^{\text{ENA}}$ = 0.138 vs. mean *F*_ST_ = 0.136).

Overall, considering the above analysis, one can predict that the SSRs loci selected to screen the rye accessions under study are suitable for downstream genetic diversity analysis.

### Genetic diversity between cultivars and landraces

Rye samples analyzed displayed different levels of diversity (number of alleles and heterozygosity). Overall, cultivars displayed lower mean allele number (3) than landraces (4), but similar genetic diversity was observed as denoted by levels of observed heterozygosity (average H_O_Cultivars = 0.70, H_O_Landraces = 0.67, Table 3). Accordingly, Matos et al. (2001) obtained a similar genetic diversity between Portuguese rye landraces and cultivars maintained in a Portuguese germplasm. Additionally, previous studies using RAPDs (Persson et al., 2001) and allozyme markers (Persson and von Bothmer, 2002, 2000) on Northern Europe rye showed that landraces and cultivars maintain roughly the same levels of genetic diversity. Overall, rye cultivars analyzed displayed similar genetic diversity as landraces, thus showing an unexpected absence of reduction in genetic diversity with increased improvement level, as recently reported for rye (Parat et al., 2016).

When narrowing analysis to landraces alone, *ex situ* collections revealed less allele per locus (3) than *in situ* (5) although showing similar heterozygosity (H_O_ *ex situ* = 0.67 vs. H_O_ *in situ* = 0.68, Table 3). This result is rather surprising, since generally higher genetic diversity would be expected within *in situ* compared to *ex situ* collections (e.g., Hou et al., 2012; Andrianasolo et al., 2013). In this study, *ex situ* and *in situ* accessions exhibit analogous genetic diversity levels, yet with different allelic diversity which may be indicative of a distinct subset of core alleles since *ex situ* are maintained under a steady environment without any selective pressure while *in situ* accessions are selected and grown by farmers in a subsistence agriculture context, being adapted under specific environments.

### Population structure

AMOVA showed that the majority of the genetic diversity lies within rye accessions with only little additional diversity present among groups or within groups. The large proportion of diversity found within accessions for the two types of groupings (cultivars vs. landraces; *ex situ* vs. *in situ*) suggests a high gene flow between accessions, which may be attributed to the wind-pollinated reproduction of rye allied with its outcrossing habit. One can therefore depict that rye-breeding system plays an important role in driving the generally observed high diversity within-accessions. Outcrossing plant species tend to have higher genetic variation within-populations, whereas selfing species or species with a mixed mating system are often genetically less variable (Nybom, 2004). Since rye is an outbreeder, negative to low inbreeding coefficients (*F*) were expected, which is in agreement with a recent rye study using SNPs (Hagenblad et al., 2016) but contrariwise to a SSRs study (Parat et al., 2016), where most populations displayed positive *F*-values which can be recorded in outcrossing populations with a strong population substructure. In the present study, low to moderate population differentiation among cultivars/*ex situ* was observed confirming the assumption of considerable population structure within cultivars and *ex situ* collection, which display steady population configuration as they are sequentially conserved in gene banks. Instead, *in situ* collections showed no population differentiation (low *F*_ST_), which may be suggestive of high gene flow between *in situ* populations from Northeast Portugal and/or an evidence of a single recent genetic source.

Generally, wind-pollinated species require a great isolation distance, since its windborne pollen may travel reasonably large distances, while in insect-pollinated species distances are related to insect activity (Richards, 1986). The low correlation between genetic and geographic distances obtained, is in agreement with earlier analysis (Hagenblad et al., 2016), and can be explained considering that pollen transfer between cultivated rye fields may easily occur as a result of its wind-pollination mode thus requiring relatively high isolation distances as established for other cross-pollinated crops (e.g., 1000–1600 m for cabbage, cauliflower). This lack of correlation between genetic and geographic distances is also in accordance with previous assumptions that attribute both temporal and ecological isolation for shaping rye's genetic diversity (Ma et al., 2004) in deterrence to spatial or geographic isolation. Moreover, individual-based clustering methods (STRUCTURE and DAPC) applied to our data highlights that genetic diversity scattering does not follow a geographic trend, regardless of being cultivars or landraces. Previous studies support the lack of clear structuring of the distribution of genetic diversity in different rye accessions depicted from geography, by using classical (i.e., allozymes, Persson and von Bothmer, 2000, 2002; RAPDs, Persson et al., 2001; AFLPs, Chikmawati et al., 2005, 2012; SSRs, Akhavan et al., 2010; Targońska et al., 2016) and modern (DaRT, Bolibok-Brągoszewska et al., 2014) molecular marker systems as well as organellar genome diversity analysis (Isik et al., 2007). However, a recent study using SNPs revealed a clustering of European landraces according to its geographic origins (Hagenblad et al., 2016). Likewise, Parat et al. (2016) managed to obtain two main subgroups indicating a differentiation according to both geography and end use, which can be described as “southern European forage rye” vs. “northern European grain rye.” In our data, despite no structuring was depicted along a geographical array, STRUCTURE analysis revealed two main subgroups indicating a differentiation between cultivars/*ex situ* with *in situ*/rye's crop wild relative. Genetic structuring between rye cultivars and landraces has been reported earlier (Persson and von Bothmer, 2000; Bolibok-Brągoszewska et al., 2014; Targońska et al., 2016), evidencing a genetic diversity assortment according to breeding status. In our data, population structuring obtained highlights a similar allelic diversity between *in situ* collections and *S. strictum*, while cultivars and *ex situ* collections do not seem to share alleles with CWR. Likewise, a multivariate approach using DAPC show that cultivars exhibit shared allelic diversity with *ex situ* accessions while *in situ* accessions might retain allelic diversity similar with the rye CWR (*K* = 2) or not (*K* = 3). Population structuring of cultivars with *ex situ* collections is further reinforced by the UPGMA analysis, which also highlights a highly supported clade consisting of *in situ* collections with the rye CWR. Altogether, our data provides evidences of cultivars and *ex situ* collections harboring a different genetic diversity in contrast to *in situ* accessions. As *ex situ* accessions offers a static genetic snapshot, reflecting a population's adaptation to environmental conditions where they were collected, and considering that cultivars are a result of a controlled breeding process, a common gene pool can be depicted between cultivars and *ex situ* accessions here analyzed from eight European regions. Particularly, *in situ* populations screened in our study are originated from the region of excellence for rye production in Portugal, with diversity being preserved under farmer management. A recent study using dominant markers in three regional populations from the Northern Portugal shows the clustering of Portuguese populations in a different set than other rye cultivars, i.e., “Imperial,” “Dankowskie Zlote,” and the Portuguese cultivar “Alvão” (Santos et al., 2016), which is in accordance to our results. Considering the dissimilar population structuring of these Portuguese *in situ* populations with cultivars and *ex situ* collections, it cannot be ruled out that the region studied may be an hotspot of rye genetic diversity yet to be explored, and thus can provide valuable knowledge about genetic diversity resulting as part of the selection process adopted by local farmers through their agricultural practices.

### *In situ* collections as a venue to a new genetic diversity: portuguese accessions as a case study

Northeast Portuguese landraces have always been of great importance for local farmers, yet few studies have been performed for addressing its genetic diversity and population structure (Matos et al., 2001; Ribeiro et al., 2012; Santos et al., 2016). Adding other accessions with different geographic origins allows performing a comprehensive assessment of the genetic diversity of Portuguese landraces. Parat et al. (2016) by studying weedy, forage and grain ryes, which included a Portuguese forage landrace, determined a high fragmentation of membership coefficients in STRUCTURE analysis, which reflects a high diversity within Portuguese accessions. Indeed, our results support this former finding, with *in situ* Portuguese accessions displaying a high fragmentation with cultivars and *ex situ* collections. Interestingly, two *ex situ* accessions, “Sved” from Sweden and “Riodeva” from Spain, grouped along *in situ* accessions, as depicted by both UPGMA and model-based clustering (STRUCTURE and DAPC, *K* = 3) analysis. Interestingly, one cultivar (“Kungs II”) and one *ex situ* accession (“R780Spain”) originated from similar geographical regions as “Sved” and “Riodeva,” respectively, displayed a distinct genetic diversity from the former accessions and with the Portuguese *in situ* populations, thus highlighting the genetic distinctiveness of such rye accessions.

“Riodeva” is a Spanish rye inbred line resulting from a selection of a local landrace of Riodeva region (Lacadena et al., 1969), further bred over 30 generations of selfing (Gallego and Benito, 1997). In our study, admixture of “Riodeva” with Portuguese *in situ* populations was disclosed, in contrary with recent data using dominant markers (Santos et al., 2016), which showed no clustering of “Riodeva” with three regional Northern Portuguese populations. In our study, notwithstanding “Riodeva” displayed a lower mean number of alleles (Supplementary Table S3) comparing to the other rye accessions screened, a higher genetic diversity was obtained with the codominant markers used. “Riodeva” inbred-line has been used as a control for aluminum (Al) susceptibility (Gallego and Benito, 1997) and studies on rye aluminum tolerance have included this accession (e.g., De Sousa et al., 2016). Therefore, admixture between Portuguese *in situ* accessions with “Riodeva” can only be related to an ancient genetic diversity that remained even after the selfing process or, to some extent, to a potential relation with Al sensitivity/tolerance in acidic soils, since rye is one of the most tolerant cereals to Al-stress, with Portuguese *in situ* populations could hold different Al-tolerance behavior yet to be uncovered. Nevertheless, further genomic studies will be needed to disclose genetic background shared between Portuguese *in situ* populations and “Riodeva,” along with the characterization of Al-tolerance behavior.

“Sved” accession is a Swedish rye landrace that clustered with another accession (also from Finnmarken, on the border between Norway and Sweden) in a way distinct from all other Scandinavian and European rye landraces (Hagenblad et al., 2016), demonstrating to be a distinct genotype not found earlier in other rye landraces, including those from the same geographical provenance. Hagenblad et al. (2016) linked its distinctiveness with historical human migrations, since Finnish farmers settled after leaving their native country in the sixteenth century (Ahokas, 2008). Considering Portuguese historic trading markets it cannot be ruled out a scenario of multiple rye introductions into Portuguese territory, especially from Northern Europe (i.e., Sweden). Indeed, historical records evidence rye grain being conveyed from Sweden to Portugal in the late eighteenth century (Ojal and Karvonen, 2012). Thus, the observed genetic similarity of a Swedish landrace with Portuguese accessions could be ascribed to a historical context. Overall, *in situ* accessions displayed a genetic kinship with a distinct landrace genotype (“Sved”) along with rye CWR, highlighting a hidden diversity on Portuguese rye gene pool yet to be uncovered.

### Rye as a rediscovered crop: implications to genetic diversity

In cross-fertilized species like rye, open-pollinated varieties (OPVs) constitute panmictic populations harboring high levels of genetic variation in their genetic build-up (Geiger and Miedaner, 2009). Moreover, improved varieties are grown in relatively uniform agricultural environments, which tend to narrow its genetic pool. Therefore, a high phenotypic variation exhibited by improved varieties might not always be a good predictor of the extent of their genetic variation (McCouch, 2004), and to surpass this concern both *in situ* and *ex situ* approaches are used to conserve the genetic diversity (Gepts, 2006). Our study shows unequivocally that *ex situ* collections display a similar genetic architecture with cultivars, sharing genetic material in a great extent with “Petkus,” one of rye's heterotic pool (Hepting, 1978). Considering that not all parental lines could be uncovered from the cultivars used in our study, and that, as far as we know, none of the used cultivars have in its pedigree “Carsten” as a parental line, we can only infer about “Petkus” genetic pool. As such, *ex situ* collections screened do not present an effective alternative for supplementing “Petkus” pool. In contrast, *in situ* Portuguese collections displayed a significant different genetic diversity than cultivars, including “Petkus,” thus being surprisingly distinctive genotypes from both cultivars and *ex situ* collections. Thus, Portuguese rye gene pool will be important for identifying new useful alleles that are linked to local adaptive processes and to its ends use, either forage or grain. This is an important finding as it sheds light onto new rye genotypes that remain to be uncovered and that could be useful for incrementing “Petkus” genetic pool. A recent study in wheat genetic diversity uncovers a pool of regional divergence, and highlights the need to increase regional breeding programs for the maintenance of crop diversity, rather than consolidation of commercial breeding alone (Novoselović et al., 2016). It is unquestionable that conservation of agricultural *in situ* genetic resources provide the genetic building blocks to improve plant varieties, and our findings unfold new *in situ* resources that will boost the improvement of new rye varieties delivering innovative information to rye breeders.

### On farm conservation: a growing importance for crop diversity

Conservation of PGR through *ex situ* and *in situ* strategies have been implemented worldwide, yet in the last decades, there has been a growing interest in on farm conservation of landraces highlighted in the Convention of Biological Diversity (CBD), Agenda 21, and the International Treaty on Plant Genetic Resources for Food and Agriculture (ITPGRFA), emphasizing the importance of on farm conservation as an essential component of sustainable agriculture. By assessing *in situ* genetic diversity one can determine which landraces may hold “new” genetic variation that could be useful to supplement crop cultivars diversity, in traits with agronomical importance (i.e., abiotic and biotic stress). Such genetic diversity is generally concentrated in centers of diversity as well as on farm conditions since landraces structure and dynamics result from both natural and human selection (Gepts, 2006). Such rich agro-historical heritage requires conservation policies to preserve the management of landraces in farmers' fields where they originated, with the aim of maintaining the evolutive processes. The outcome of on farm conservation can be conceptualized as “evolutionary service” to agricultural and food systems, and to function, it depends on farmers' preferences, knowledge, management, practices, and social organization.

On farm conservation of local landraces, particularly those found in our study, reflects a specific case, since we studied landraces not listed at both national and international gene banks. As such, we believe that the first intervention toward a conservation protocol is to implement a new national seed policy on landraces, which usually favors only varieties that are distinct, uniform and stable, discouraging the use of more heterogeneous, variable landraces. In agricultural systems as the one practiced in Northern Portugal rye fields, farmers typically save seed from one season to the next and may share seed with other farmers, being seed sourcing embedded in well-structured traditional systems with rules and expectations based on family and local social networks (Veteläinen et al., 2009). As such, on farm conservation protocols should be compatible with improved livelihoods and well-being among farmers who conserve such landraces, by incrementing ecosystem services at regional and national level and by giving public benefits as a stimulus to promote specialized or novel marketing niches based on landraces and on local cultural heritage. Such landraces should be maintained as *in situ* genetic reserves and, besides *ex situ* conservation at national and international gene banks, an inventory periodically updated should be pursued to monitor on farm maintenance of landrace diversity.

Overall, our study successfully illustrates the significance of comparing the genetic diversity and structure of *ex situ* and *in situ* samples, along rye cultivars thus highlighting *in situ* collections from Northeast Portugal as new genetic resources being distinct genotypes to those reported for rye *ex situ* and cultivars. Identification of alleles/genes underlying such distinctive diversity would be of utmost importance for determining their usefulness for incorporating future rye breeding programs and to additionally propose on farm conservation policies at national level.

## Author contributions

FM, AM, HO, and WV designed the research. FM carried out molecular work. FM, PV, and AB analyzed the data. FM wrote the manuscript and all authors improved upon versions. All authors read and approved the final manuscript.

### Conflict of interest statement

The authors declare that the research was conducted in the absence of any commercial or financial relationships that could be construed as a potential conflict of interest.
