# Supplementary material for: Genetic Distinctiveness of Rye In situ Accessions from Portugal Unveils a New Hotspot of Unexplored Genetic Resources
Source: Front Plant Sci. 2016 Aug 31;7:1334. doi: 10.3389/fpls.2016.01334 (PMC5006150; doi:10.3389/fpls.2016.01334)
Supplement: Supplementary file 1 [file Table1.pdf]

Supplementary Material

Genetic distinctiveness of rye *in situ* accessions from Portugal unveils a new hotspot of unexplored genetic resources

Filipa Monteiro*, Patrícia Vidigal, André B. Barros, Ana Monteiro, Hugo R. Oliveira and Wanda Viegas

***Correspondence**: **Filipa Monteiro** fmonteiro@isa.ulisboa.pt

Supplementary Table S1. Loci used to screen 29 rye populations. Primers sequences, multiplexing scheme and putative function for EST-SSRs are provided. EST-SSRs are highlighted in bold lettering; while with the remaining loci are gSSR.

Locus	Repeat motif	Chromosome location	Primers (5'-3')	Tailed Primer	Size range (Expected Size)	Reference	Multiplex	Putative function
**SCM152**	(AG)$_{7}$	unknown	F: CGGAGCAGCAGAGCAAGAGA<br>R: GTTTCTTATGTAGCCGAGGATGGTGAGC	D1	319-375 (**171**)	1	A	Hydrophobic polypeptide
**SCM66**	(CCG)$_{7}$	4R	F:CCCAATTCCCCACGCGCAAATC<br>R: GTTTCTTATGTAGCCGAGGATGGTGAGC	D2	157-166 (**168**)	1		Ribonucleotide reductase R2
SCM86	(GT)$_{20}$	7R	F: CAGATAGATGGGTGTTGTGCG<br>R: GTTTCTTCTCTTCTCGACATCCACACTCC	D3	94-122 (**117**)	2		-
SCM138	(AC)$_{23}$	5RS	F: ATAGCCGCAGATGGTTGAGGAC<br>R: GTTTCTTGAGAAGTCTACAAATCAAGGGGGC	D4	171-193 (**188**)	2		-
**SCM63**	(CCG)$_{5}$	unknown	F: CGACTTCGAGGGCAGGAATGA<br>R: GTTTCTTATCCCGGGGATGAAGTGCAG	D1	197-257 (**147**)	1	B	Putative β-galactosidase
SCM75	(CA)$_{7}$(CT)$_{15}$…(CA)$_{10}$	2R	F: TTTTCTATCTCAGCGATTCATGC<br>R: GTTTCTTTCCTGAGATCAAGTGCGTGTG	D2	180-200 (**191**)	2		-
SCM28	(GT)$_{26}$	unknown	F: CTGGTCCTGGTCTGGTGGGTC<br>R: GTTTCTTCGCATCGGGTGTGTCGCATAC	D3	126-162 (**159**)	2		-
**SCM164**	(CCT)$_{5}$	unknown	F: TCGATGGGCTCTGTCCTGT<br>R: GTTTCTTACAAAGTTTGTGGCGGTTCTG	D4	140-159 (**145**)	1		Putative double strand break repair protein
SCM43	(GT)$_{11}$	unknown	F: CTAGGGGATTACAGGGAGGGCA	D1	88-116 (**100**)	2	C	-

			R: GTTTCTTGTTCCCTTGTCCTACTCGTTACCG					
SCM9	$(GT)_8$	1RS	F: TGACAACCCCCTTTCCCTCGT<br>R: GTTTCTTTCATCGACGCTAAGGAGGACCC	D2	176-226 (**220**)	2		-
SCM39	$(GT)_8(GC)_6(GT)_{53}$	unknown	F: GACCTCAGTGGAGCCTCTAGGT<br>R: GTTTCTTGGACATCTGCCGTGACAATACC	D3	177-199 (**230**)	2		-
SCM180	$(GA)_6(GA)_7$	6RL	F: GTTTCGTCCCCGTTGCCATC<br>R: GTTTCTTACGTGTCGCTTTCCATTGCCC	D4	(**140**)	2		-
**SCM98**	$(CTG)_5$	unknown	F:TGCTGCTCATTGCTACTGTCA<br>R: GTTTCTTCGATCCAGGATAACGGCTACA	D1	137-146 (**142**)	1	D	High mobility group protein HMGb1
**SCM113**	$(GTC)_5$	unknown	F: CCACGCCATGTCCATCTCCA<br>R: GTTTCTTGCATGCGGAACTCCTCCAT	D2	194 (**194**)	1		Alcohol dehydrogenase-like protein
**SCM166**	$(CCT)_5$	unknown	F: TCTCCGAGAGGCCAAGCAGAAA<br>R: GTTTCTTATGGCGACCACCCCGAATC	D3	194-245 (**125**)	1		Apospory-associated protein C-like
SCM2	$(GT)_{10}$	6RL	F: GATGACTATGACTACCAGGATGAA<br>R: GTTTCTTGGAGTGAGAAGGCCGAGAAG	D4	112-120 (**113**)	2		-

Following Culley et al. (2013), D1 (6-FAM): M13 (-21), 5'-TGTAAAACGACGGCCAGT-3'; D2 (NED,): T7term, 5'-CTAGTTATTGCTCAGCGGT-3'; D3 (VIC): M13modA, 5'-TAGGAGTGCAGCAAGCAT-3'; D4 (PET): M13modB, 5'-CACTGCTTAGAGCGATGC-3'. 1- Hackauf and Wehling (2002); 2- Saal & Wricke,1999.
