# Supplementary material for: Genetic Distinctiveness of Rye In situ Accessions from Portugal Unveils a New Hotspot of Unexplored Genetic Resources
Source: Front Plant Sci. 2016 Aug 31;7:1334. doi: 10.3389/fpls.2016.01334 (PMC5006150; doi:10.3389/fpls.2016.01334)
Supplement: Supplementary file 2 [file Table2.pdf]

Supplementary Material

Genetic distinctiveness of rye *in situ* accessions from Portugal unveils a new hotspot of unexplored genetic resources

Filipa Monteiro*, Patrícia Vidigal, André B. Barros, Ana Monteiro, Hugo R. Oliveira and Wanda Viegas

***Correspondence**: **Filipa Monteiro** fmonteiro@isa.ulisboa.pt

Supplementary Table S2. Hardy–Weinberg equilibrium (HWE) test for each locus-population combination using GenePop v4.5. Statistical significance was assessed by running 10,000 iterations Monte Carlo Markov Chain (MCMC) test. *p*-values were corrected by multiple comparisons applying a sequential Bonferroni correction ($p<0.00012$, [0.05/406]). $p<0.05$, dark blue; $p<0.01$, blue; $p<0.00012$, light blue.

		SCM138	SCM152	SCM164	SCM166	SCM2	SCM39	SCM43	SCM63	SCM66	SCM75	SCM86	SCM9	SCM98	SCM28
Cultivars	Ailés	0.36	0.21	1	-	1	0.31	0.42	1	0.39	0.39	0.35	1	1	0.09
	Anton	0.52	1	1	0.11	0.43	-	1	1	0.09	0.90	0.48	-	1	1
	Dankow	0.79	0.09	0.51	0.72	0.25	0.21	0.86	1	0.09	1	0.88	0.72	0.64	0.63
	Imperial	0.42	1	0.10	0.39	0.39	0.22	0.20	-	0.09	0.48	0.58	0.69	0.64	0.58
	Kungs II	0.31	1	1	1	0.39	1	1	0.64	0.09	0.20	0.04	1	0.09	0.22
	Petkus	0.41	0.21	0.10	1	0.09	0.01	0.52	0.13	0.09	1	0.58	0.02	0.09	0.52
	Voima	0.89	0.79	1	1	0.58	0.48	0.88	0.64	0.09	1	0.33	0.06	0.09	0.75
	Pulawskie	0.34	1	1	0.58	0.08	0.03	0.31	1	0.09	0.86	0.34	0.20	0.09	0.15
	Alvão	0.43	0.52	1	0.09	0.52	0.09	0.56	0.19	1	0.86	0.03	1	0.09	0.21
ex situ accessions	Riodeva	0.09	-	0.39	-	-	-	0.48	0.09	0.09	0.08	0.09	0.09	-	1
	Sved	0.01	-	0.14	0.38	0.32	0.49	0.33	0.49	0.03	0.69	0.03	1	1	0.06
	R2136Russ	0.56	-	1	0.20	0.39	1	0.75	0.20	0.09	0.35	1	0.64	0.64	0.13
	R780Spain	0.32	1	-	1	0.39	0.09	0.34	1	0.09	1	0.64	0.79	0.09	0.63
	R2694West	1	0.86	1	0.09	1	-	0.20	1	0.09	0.66	1	0.39	0.09	0.08
	R1148Turk	0.52	0.86	0.43	0.27	0.20	0.09	0.09	1	1	0.07	0.19	0.52	1	0.75
	R1138Ital	1	0.07	0.03	1	0.09	0.03	1	0.64	0.09	0.09	0.20	0.48	0.09	1
	R1133PT	0.05	0.89	0.65	1	0.20	0.32	0.05	-	0.09	0.63	0.31	0.32	0.09	0.65
in situ accessions	SECCE1	0.83	0.00	0.00	0.23	0.62	0.18	0.03	0.00	0.59	0.00	0.14	0.56	0.80	0.03
	SECCE2	0.94	0.05	0.00	0.30	0.56	0.17	0.30	0.00	1	0.05	0.96	0.36	0.02	0.01
	SECCE3	0.80	0.22	0.23	0.15	0.02	0.04	0.09	0.00	0.03	0.32	0.31	0.66	0.23	0.54
	SECCE4	0.90	0.00	0.00	0.09	1	0.00	0.06	0.00	1	0.07	0.42	0.08	0.11	0.15
	SECCE5	0.32	0.41	0.00	0.15	1	0.39	0.24	0.00	0.64	0.13	0.25	0.38	0.19	0.06
	SECCE6	0.26	0.02	0.00	1	0.59	0.20	0.61	0.00	1	0.03	0.93	0.00	0.11	0.00
	SECCE7	0.15	0.59	0.00	1	0.33	0.40	0.91	0.00	0.00	0.00	0.00	0.04	0.00	0.33
	SECCE8	0.07	0.01	0.17	0.57	0.46	1	0.36	0.00	0.00	0.00	0.04	0.00	0.36	0.08
	SECCE9	0.98	0.62	0.00	1	0.40	0.23	0.20	0.00	0.00	0.03	0.00	0.00	0.27	0.01
	SECCE10	0.37	0.32	0.00	1	0.32	0.01	0.09	0.00	0.00	0.11	0.31	0.02	1	0.21
	SECCE11	0.21	0.00	0.01	1	1	0.12	0.15	0.02	0.00	0.02	0.00	0.02	0.62	0.01
	Secale strictum	0.13	-	0.13	0.13	0.13	-	1	0.13	0.13	-	1	0.43	0.13	0.13
