# Supplementary material for: Genetic Distinctiveness of Rye In situ Accessions from Portugal Unveils a New Hotspot of Unexplored Genetic Resources
Source: Front Plant Sci. 2016 Aug 31;7:1334. doi: 10.3389/fpls.2016.01334 (PMC5006150; doi:10.3389/fpls.2016.01334)
Supplement: Supplementary file 3 [file Table3.pdf]

frontiers

Supplementary Material

Genetic distinctiveness of rye *in situ* accessions from Portugal unveils a new hotspot of unexplored genetic resources

Filipa Monteiro*, Patrícia Vidigal, André B. Barros, Ana Monteiro, Hugo R. Oliveira and Wanda Viegas

***Correspondence**: **Filipa Monteiro** fmonteiro@isa.ulisboa.pt

Supplementary Table S3. Genetic diversity analysis by population. For each population, genetic diversity indices was assessed by expected heterozygosity (He) and observed heterozygosity (H_O) with corresponding standard deviation (SD) values, inbreeding/fixation coefficient (*F*), and mean alleles per locus (N_a), and null allele frequency as determined by FreeNA. Data are provided by total SSRs, gSSR and EST-SSRs, following by the grouping scheme adopted, with sample size (N): cultivars, *ex situ* and *in situ* collections, which collectively are referred as landraces and total sampling (i.e. sampled accessions including *S. strictum*).

All SSRs

Population	Sample size	Loci typed	He	He SD	H_O	H_O SD	Na	Na SD	*F*	Frequency Null Alleles (>0.20)
01. Aile	6	14	0.55	0.07	0.61	0.05	2.93	1.39	-0.24	-
02. Antoninskie	6	14	0.45	0.07	0.57	0.05	2.80	1.37	-0.36	-
03. Dankowskie zlote	6	14	0.58	0.06	0.62	0.05	3.20	1.21	-0.14	0.25 (SCM152)
04. Imperial	6	14	0.56	0.06	0.69	0.05	2.67	0.98	-0.37	-
05. Kungs II	6	14	0.55	0.05	0.69	0.05	2.93	1.10	-0.39	-
06. Petkus	6	14	0.62	0.05	0.70	0.05	3.40	1.40	-0.26	-
07. Voima	6	14	0.64	0.06	0.79	0.04	3.40	1.40	-0.38	-
08. Pulawskie	6	14	0.63	0.05	0.69	0.05	3.53	1.36	-0.22	-
09. Alvão	6	14	0.57	0.06	0.53	0.05	3.33	1.50	-0.02	0.21 (SCM39)
		**Avg cultivars**	0.57	0.06	0.65	0.05	3.13	1.30	-0.26	
		**SD cultivars**	0.02	0.00	0.03	0.00	0.10	0.06	0.04	
10. Sved	8	14	0.48	0.05	0.62	0.04	2.60	0.91	-0.37	**Frequency Null Alleles (>0.20)**

11. Riodeva	6	14	0.35	0.08	0.56	0.05	1.93	1.03	-0.76	-
12. R2136Russ	6	14	0.55	0.06	0.64	0.05	3.00	1.20	-0.29	-
13. R780Spain	6	14	0.56	0.06	0.74	0.05	3.07	1.28	-0.45	-
14. R2694West	6	14	0.53	0.06	0.66	0.05	2.93	1.22	-0.36	-
15. R1148Turkey	6	14	0.56	0.06	0.54	0.05	3.27	1.28	-0.04	0.22 (SCM75), 0.25 (CM39)
16. R1138Italy	6	14	0.58	0.05	0.66	0.05	3.00	1.07	-0.25	0.25 (SCM164), 0.25 (SCM39)
17. R1133PT	6	14	0.57	0.07	0.56	0.05	2.93	1.16	-0.10	0.21 (SCM138)
**Avg *ex situ* collections**			0.52	0.06	0.62	0.05	2.84	1.14	-0.33	
**SD *ex situ* collections**			0.03	0.00	0.02	0.00	0.15	0.05	0.08	**Frequency Null Alleles (>0.20)**
18. SECCE1	16	14	0.66	0.06	0.60	0.03	5.87	2.95	0.05	-
19. SECCE2	16	14	0.65	0.06	0.62	0.03	5.67	2.44	0.02	0.21 (SCM63)
20. SECCE3	16	14	0.69	0.06	0.64	0.03	6.00	2.59	0.05	-
21. SECCE4	16	14	0.65	0.06	0.60	0.03	5.40	2.23	0.03	-
22. SECCE5	16	14	0.66	0.06	0.63	0.03	5.67	2.85	0.00	0.25 (SCM63)
23. SECCE6	16	14	0.66	0.06	0.63	0.03	5.87	2.80	0.00	-
24. SECCE7	16	14	0.59	0.06	0.66	0.03	4.93	2.58	-0.17	-
25. SECCE8	16	14	0.62	0.06	0.64	0.03	5.47	2.77	-0.04	-
26. SECCE9	16	14	0.56	0.05	0.68	0.03	3.93	1.87	-0.27	-
27. SECCE10	16	14	0.58	0.06	0.63	0.03	4.40	1.84	-0.15	0.26 (SCM63)
28. SECCE11	16	14	0.56	0.05	0.69	0.03	4.13	2.10	-0.30	-
**Avg *in situ* collections**			0.63	0.06	0.64	0.03	5.21	2.46	-0.07	
**SD *in situ* collections**			0.01	0.00	0.01	0.00	0.22	0.12	0.04	
**Avg Landraces**			0.58	0.06	0.63	0.04	4.21	1.90	-0.18	
**SD Landraces**			0.02	0.00	0.01	0.00	0.31	0.17	0.05	
29. *Secale strictum*	5	14	0.41	0.07	0.68	0.05	1.93	0.96	-0.85	-
**Avg Total**			0.57	0.06	0.64	0.04	3.80	1.68	-0.23	
**StDv Total**			0.01	0.00	0.01	0.00	0.23	0.12	0.04	

gSSRs										
**Population**	**N**	**Loci**	**He**	**He SD**	**H_O**	**H_O SD**	**Na**	**Na SD**	***F***	**Frequency Null Alleles (>0.20)**
01. Aile	6	8	0.70	0.04	0.73	0.06	3.75	1.28	-0.14	-
02. Antoninskie	6	8	0.47	0.11	0.60	0.07	3.13	1.64	-0.39	-
03. Dankowskie zlote	6	8	0.70	0.04	0.74	0.06	4.00	0.93	-0.16	-
04. Imperial	6	8	0.69	0.03	0.81	0.06	3.25	0.71	-0.28	-
05. Kungs II	6	8	0.63	0.06	0.69	0.07	3.50	1.07	-0.21	-
06. Petkus	6	8	0.69	0.04	0.75	0.06	4.13	1.36	-0.20	-
07. Voima	6	8	0.79	0.02	0.93	0.04	4.25	1.04	-0.30	-
08. Pulawskie	6	8	0.74	0.03	0.73	0.06	4.25	1.16	-0.06	-
09. Alvao	6	8	0.71	0.04	0.60	0.07	4.25	1.39	0.08	0.21 (SCM39)
**Avg cultivars**			0.68	0.04	0.73	0.06	3.83	1.17	-0.18	
**SD cultivars**			0.03	0.01	0.03	0.00	0.15	0.09	0.05	**Frequency Null Alleles (>0.20)**
10. Sved	8	8	0.57	0.04	0.70	0.06	3.13	0.83	-0.33	-
11. Riodeva	6	8	0.44	0.10	0.69	0.07	2.25	1.16	-0.73	-
12. R2136Russ	6	8	0.66	0.06	0.73	0.06	3.63	1.19	-0.24	-
13. R780Spain	6	8	0.69	0.04	0.85	0.05	3.75	1.16	-0.39	-
14. R2694West	6	8	0.59	0.09	0.62	0.07	3.13	1.25	-0.18	-
15. R1148Turkey	6	8	0.69	0.07	0.58	0.07	3.75	1.04	0.12	0.22 (SCM75), 0.25 (SCM39)
16. R1138Italy	6	8	0.64	0.05	0.73	0.06	3.38	1.06	-0.25	0.25 (SCM39)
17. R1133PT	6	8	0.72	0.02	0.60	0.07	3.63	0.74	0.08	0.21 (SCM138)
**Avg *ex situ* collections**			0.62	0.06	0.69	0.06	3.33	1.05	-0.24	
**SD *ex situ* collections**			0.03	0.01	0.03	0.00	0.18	0.06	0.10	**Frequency Null Alleles (>0.20)**
18. SECCE1	16	8	0.74	0.05	0.70	0.04	6.63	2.20	0.03	-
19. SECCE2	16	8	0.71	0.06	0.73	0.04	6.50	1.60	-0.06	-
20. SECCE3	16	8	0.78	0.03	0.75	0.04	7.13	1.96	0.00	-
21. SECCE4	16	8	0.72	0.05	0.67	0.04	6.50	1.31	0.02	-
22. SECCE5	16	8	0.73	0.05	0.69	0.04	6.50	2.20	0.00	-
23. SECCE6	16	8	0.74	0.05	0.71	0.04	6.75	2.19	-0.01	-
24. SECCE7	16	8	0.70	0.04	0.82	0.03	5.75	2.43	-0.24	-

25. SECCE8	16	8	0.73	0.05	0.73	0.04	6.88	2.53	-0.06	-
26. SECCE9	16	8	0.63	0.04	0.80	0.04	4.38	1.41	-0.34	-
27. SECCE10	16	8	0.64	0.03	0.71	0.04	4.50	1.41	-0.15	-
28. SECCE11	16	8	0.64	0.05	0.82	0.03	4.88	2.17	-0.34	-
**Avg *in situ* collections**			0.70	0.04	0.74	0.04	6.03	1.95	-0.10	
**SD *in situ* collections**			0.02	0.00	0.02	0.00	0.30	0.13	0.04	
**Avg Landraces**			0.67	0.05	0.72	0.05	4.89	1.57	-0.16	
**SD Landraces**			0.02	0.00	0.02	0.00	0.37	0.13	0.05	
29. *Secale strictum*	5	8	0.42	0.10	0.65	0.08	2.13	1.25	-0.72	-
**Avg Total**			0.67	0.05	0.72	0.05	4.47	1.44	-0.19	
**SD Total**			0.02	0.00	0.01	0.00	0.27	0.10	0.04	

**EST-SSRs**										
**Population**	**N**	**Loci**	**He**	**He SD**	**H_O**	**H_O SD**	**Na**	**Na SD**	***F***	**Frequency Null Alleles (>0.20)**
01. Aile	6	6	0.38	0.10	0.48	0.08	2.00	0.82	-0.39	-
02. Antoninskie	6	6	0.39	0.07	0.50	0.08	2.43	0.98	-0.33	-
03. Dankowskie zlote	6	6	0.44	0.09	0.48	0.08	2.29	0.76	-0.11	0.25 (SCM152)
04. Imperial	6	6	0.40	0.11	0.55	0.08	2.00	0.82	-0.51	-
05. Kungs II	6	6	0.46	0.08	0.69	0.07	2.29	0.76	-0.62	-
06. Petkus	6	6	0.55	0.09	0.64	0.07	2.57	0.98	-0.33	-
07. Voima	6	6	0.48	0.09	0.64	0.07	2.29	0.95	-0.49	-
08. Pulawskie	6	6	0.50	0.09	0.64	0.07	2.71	1.11	-0.43	-
09. Alvao	6	6	0.42	0.08	0.45	0.08	2.29	0.76	-0.15	-
**Avg cultivars**			0.45	0.09	0.56	0.08	2.32	0.88	-0.37	
**SD cultivars**			0.02	0.00	0.03	0.00	0.08	0.04	0.06	**Frequency Null Alleles (>0.20)**
10. Sved	8	6	0.37	0.08	0.52	0.07	2.00	0.58	-0.42	-
11. Riodeva	6	6	0.24	0.11	0.40	0.08	1.57	0.79	-0.82	-
12. R2136Russ	6	6	0.43	0.10	0.55	0.08	2.29	0.76	-0.36	-
13. R780Spain	6	6	0.43	0.09	0.62	0.07	2.29	0.95	-0.54	-

14. R2694West	6	6	0.48	0.09	0.69	0.07	2.71	1.25	-0.58	-
15. R1148Turkey	6	6	0.43	0.10	0.50	0.08	2.71	1.38	-0.26	-
16. R1138Italy	6	6	0.51	0.09	0.57	0.08	2.57	0.98	-0.24	0.25 (SCM164)
17. R1133PT	6	6	0.40	0.12	0.50	0.08	2.14	1.07	-0.40	-
**Avg *ex situ* collections**			0.41	0.10	0.54	0.07	2.29	0.97	-0.45	
**SD *ex situ* collections**			0.03	0.00	0.03	0.00	0.14	0.09	0.07	**Frequency Null Alleles (>0.20)**
18. SECCE1	16	6	0.58	0.12	0.50	0.05	5.00	3.61	0.07	-
19. SECCE2	16	6	0.58	0.11	0.48	0.05	4.43	2.64	0.12	0.21 (SCM63)
20. SECCE3	16	6	0.58	0.11	0.50	0.05	4.57	2.51	0.11	-
21. SECCE4	16	6	0.58	0.11	0.52	0.05	4.00	2.31	0.03	-
22. SECCE5	16	6	0.58	0.12	0.54	0.05	4.71	3.35	0.00	-
23. SECCE6	16	6	0.58	0.12	0.53	0.05	4.71	2.98	0.03	-
24. SECCE7	16	6	0.47	0.10	0.47	0.05	4.00	2.58	-0.07	-
25. SECCE8	16	6	0.49	0.09	0.49	0.05	3.71	1.80	-0.02	0.25 (SCM63)
26. SECCE9	16	6	0.48	0.11	0.53	0.05	3.43	2.30	-0.17	-
27. SECCE10	16	6	0.51	0.12	0.52	0.05	4.14	2.27	-0.14	0.26 (SCM63)
28. SECCE11	16	6	0.46	0.08	0.54	0.05	3.29	1.80	-0.25	-
**Avg *in situ* collections**			0.54	0.11	0.51	0.05	4.18	2.56	-0.03	
**SD *in situ* collections**			0.02	0.00	0.01	0.00	0.17	0.17	0.04	
**Avg Landraces**			0.48	0.10	0.53	0.06	3.38	1.89	-0.21	
**SD Landraces**			0.02	0.00	0.01	0.00	0.25	0.21	0.06	
29. *Secale strictum*	5	6	0.40	0.10	0.71	0.08	1.71	0.49	-1.00	-
**Avg Total**			0.46	0.10	0.53	0.06	2.95	1.52	-0.28	
**SD Total**			0.02	0.00	0.01	0.00	0.19	0.17	0.05	
