# Supplementary material for: Genetic Distinctiveness of Rye In situ Accessions from Portugal Unveils a New Hotspot of Unexplored Genetic Resources
Source: Front Plant Sci. 2016 Aug 31;7:1334. doi: 10.3389/fpls.2016.01334 (PMC5006150; doi:10.3389/fpls.2016.01334)
Supplement: Supplementary file 4 [file Table4.pdf]

Supplementary Material

Genetic distinctiveness of rye *in situ* accessions from Portugal unveils a new hotspot of unexplored genetic resources

Filipa Monteiro*, Patrícia Vidigal, André B. Barros, Ana Monteiro, Hugo R. Oliveira and Wanda Viegas

***Correspondence**: **Filipa Monteiro** fmonteiro@isa.ulisboa.pt

Supplementary Table S4. Pairwise F_{ST} (lower-left matrix) F_{ST}^{ENA} (upper-right matrix) between all populations.

F_{ST}^{ENA} (columns); F_{ST} (rows)

	Aile	Anton	Dankow	Imperial	Kungs	Petkus	Voima	Pulaw	Alvao	Riodeva	Sved	R2136Russ	R780Spain	R2694West	R1148Turk	R1138Ital	R1133PT	SECCE1	SECCE2	SECCE3	SECCE4	SECCE5	SECCE6	SECCE7	SECCE8	SECCE9	SECCE10	SECCE11	S.strictum
Aile	0.00	0.21	0.15	0.17	0.19	0.13	0.12	0.12	0.06	0.27	0.23	0.15	0.15	0.17	0.09	0.11	0.12	0.12	0.14	0.09	0.11	0.11	0.10	0.12	0.09	0.17	0.14	0.15	0.34
Anton	0.21	0.00	0.15	0.16	0.28	0.19	0.15	0.13	0.15	0.32	0.19	0.12	0.18	0.14	0.11	0.21	0.18	0.21	0.20	0.15	0.18	0.18	0.13	0.16	0.12	0.17	0.16	0.14	0.35
Dankow	0.15	0.15	0.00	0.10	0.11	0.02	0.07	0.02	0.07	0.34	0.25	0.14	0.12	0.11	0.11	0.10	0.08	0.13	0.17	0.11	0.16	0.16	0.13	0.16	0.13	0.18	0.16	0.12	0.36
Imperial	0.17	0.15	0.11	0.00	0.21	0.13	0.13	0.08	0.12	0.34	0.24	0.15	0.14	0.16	0.11	0.12	0.11	0.15	0.16	0.11	0.15	0.14	0.14	0.16	0.14	0.19	0.16	0.15	0.33
Kungs	0.19	0.28	0.12	0.21	0.00	0.07	0.08	0.13	0.15	0.39	0.26	0.17	0.17	0.18	0.20	0.18	0.21	0.17	0.19	0.13	0.20	0.18	0.16	0.19	0.18	0.23	0.21	0.18	0.40
Petkus	0.13	0.19	0.02	0.13	0.07	0.00	0.07	0.07	0.07	0.34	0.22	0.13	0.14	0.12	0.10	0.08	0.12	0.10	0.13	0.09	0.15	0.14	0.11	0.15	0.12	0.17	0.15	0.12	0.33
Voima	0.13	0.15	0.07	0.13	0.08	0.07	0.00	0.08	0.06	0.29	0.20	0.08	0.08	0.05	0.09	0.09	0.08	0.10	0.11	0.07	0.12	0.10	0.08	0.12	0.08	0.15	0.13	0.10	0.28
Pulawskie	0.12	0.12	0.02	0.09	0.13	0.07	0.08	0.00	0.08	0.30	0.22	0.10	0.12	0.13	0.09	0.09	0.09	0.13	0.16	0.10	0.13	0.13	0.11	0.14	0.11	0.17	0.15	0.13	0.29
Alvao	0.07	0.14	0.07	0.12	0.15	0.07	0.06	0.07	0.00	0.28	0.21	0.11	0.08	0.12	0.07	0.07	0.08	0.09	0.12	0.08	0.10	0.09	0.07	0.11	0.07	0.14	0.11	0.09	0.32
Riodeva	0.27	0.32	0.34	0.33	0.39	0.34	0.29	0.30	0.28	0.00	0.19	0.26	0.29	0.28	0.24	0.28	0.27	0.20	0.21	0.20	0.18	0.13	0.16	0.11	0.12	0.12	0.16	0.15	0.42
Sved	0.23	0.19	0.26	0.24	0.26	0.22	0.20	0.21	0.21	0.18	0.00	0.18	0.21	0.21	0.18	0.22	0.20	0.19	0.19	0.16	0.18	0.13	0.13	0.08	0.08	0.09	0.15	0.09	0.32
R2136Russ	0.15	0.12	0.13	0.15	0.16	0.13	0.08	0.10	0.10	0.26	0.18	0.00	0.13	0.08	0.11	0.15	0.16	0.14	0.12	0.07	0.12	0.12	0.10	0.14	0.10	0.16	0.12	0.14	0.30
R780Spain	0.15	0.18	0.13	0.13	0.17	0.14	0.08	0.11	0.08	0.29	0.21	0.13	0.00	0.12	0.11	0.13	0.07	0.12	0.15	0.09	0.14	0.11	0.09	0.13	0.10	0.14	0.11	0.11	0.34
R2694West	0.17	0.15	0.12	0.16	0.17	0.12	0.05	0.13	0.11	0.29	0.21	0.08	0.13	0.00	0.11	0.10	0.13	0.14	0.15	0.10	0.16	0.14	0.11	0.14	0.09	0.16	0.14	0.13	0.32
R1148Turk	0.10	0.11	0.12	0.12	0.21	0.10	0.10	0.09	0.07	0.23	0.18	0.11	0.12	0.11	0.00	0.08	0.06	0.09	0.09	0.07	0.09	0.09	0.05	0.09	0.06	0.11	0.08	0.09	0.28
R1138Ital	0.11	0.21	0.11	0.13	0.18	0.08	0.09	0.09	0.07	0.27	0.21	0.15	0.13	0.10	0.07	0.00	0.09	0.10	0.13	0.10	0.12	0.11	0.08	0.11	0.10	0.15	0.14	0.12	0.33
R1133PT	0.12	0.17	0.09	0.11	0.22	0.12	0.09	0.09	0.07	0.26	0.20	0.16	0.06	0.13	0.07	0.08	0.00	0.10	0.14	0.11	0.10	0.10	0.09	0.11	0.08	0.10	0.09	0.08	0.32
SECCE1	0.11	0.20	0.13	0.14	0.17	0.10	0.09	0.12	0.09	0.19	0.18	0.13	0.11	0.13	0.09	0.10	0.09	0.00	0.04	0.04	0.05	0.04	0.05	0.09	0.07	0.10	0.08	0.09	0.26
SECCE2	0.13	0.19	0.17	0.16	0.18	0.13	0.10	0.15	0.12	0.20	0.19	0.12	0.15	0.15	0.08	0.13	0.13	0.04	0.00	0.02	0.02	0.04	0.04	0.09	0.08	0.10	0.08	0.12	0.22
SECCE3	0.09	0.14	0.11	0.10	0.13	0.09	0.07	0.10	0.08	0.19	0.16	0.07	0.09	0.10	0.07	0.10	0.11	0.03	0.02	0.00	0.03	0.04	0.03	0.09	0.07	0.10	0.09	0.11	0.19
SECCE4	0.11	0.18	0.17	0.15	0.19	0.14	0.11	0.12	0.10	0.17	0.18	0.11	0.13	0.16	0.09	0.13	0.11	0.05	0.02	0.03	0.00	0.02	0.03	0.07	0.07	0.08	0.08	0.10	0.22
SECCE5	0.11	0.17	0.17	0.13	0.18	0.14	0.09	0.12	0.09	0.12	0.12	0.12	0.11	0.14	0.09	0.11	0.10	0.04	0.03	0.03	0.02	0.00	0.02	0.04	0.03	0.05	0.08	0.08	0.19
SECCE6	0.10	0.13	0.14	0.14	0.16	0.11	0.08	0.11	0.07	0.15	0.13	0.10	0.09	0.11	0.06	0.09	0.09	0.04	0.04	0.03	0.03	0.02	0.00	0.04	0.04	0.07	0.07	0.07	0.23
SECCE7	0.12	0.15	0.16	0.16	0.19	0.15	0.11	0.14	0.11	0.10	0.08	0.13	0.12	0.14	0.09	0.11	0.10	0.09	0.09	0.08	0.07	0.03	0.04	0.00	0.01	0.02	0.05	0.03	0.25
SECCE8	0.09	0.11	0.13	0.13	0.17	0.12	0.07	0.10	0.07	0.11	0.08	0.10	0.09	0.09	0.06	0.10	0.08	0.07	0.08	0.07	0.07	0.03	0.04	0.01	0.00	0.03	0.05	0.03	0.21
SECCE9	0.17	0.16	0.19	0.18	0.23	0.17	0.14	0.17	0.14	0.11	0.09	0.16	0.14	0.16	0.11	0.16	0.09	0.10	0.10	0.10	0.08	0.05	0.07	0.02	0.03	0.00	0.06	0.04	0.24
SECCE10	0.14	0.15	0.16	0.15	0.20	0.14	0.12	0.14	0.11	0.16	0.14	0.11	0.10	0.14	0.08	0.14	0.08	0.07	0.08	0.09	0.08	0.08	0.07	0.05	0.05	0.06	0.00	0.05	0.27
SECCE11	0.15	0.14	0.13	0.15	0.18	0.12	0.10	0.12	0.09	0.14	0.09	0.14	0.11	0.13	0.10	0.13	0.08	0.09	0.12	0.11	0.11	0.08	0.07	0.03	0.03	0.04	0.05	0.00	0.29
S. strictum	0.35	0.35	0.36	0.33	0.40	0.33	0.28	0.29	0.31	0.42	0.32	0.29	0.34	0.32	0.27	0.32	0.31	0.24	0.21	0.19	0.21	0.18	0.23	0.25	0.20	0.24	0.26	0.29	0.00

	Fst	FstENA
Goblal Mean	**0.136**	**0.138**
Cultivars mean	**0.119**	0.119
Landrace mean	0.11	0.108
ex situ collections mear	0.163	0.165
in situ collections Mean	0.057	0.059
