# Supplementary material for: Genetic Distinctiveness of Rye In situ Accessions from Portugal Unveils a New Hotspot of Unexplored Genetic Resources
Source: Front Plant Sci. 2016 Aug 31;7:1334. doi: 10.3389/fpls.2016.01334 (PMC5006150; doi:10.3389/fpls.2016.01334)
Supplement: Supplementary file 5 [file Table5.pdf]

Supplementary Material

Genetic distinctiveness of rye *in situ* accessions from Portugal unveils a new hotspot of unexplored genetic resources

Filipa Monteiro*, Patrícia Vidigal, André B. Barros, Ana Monteiro, Hugo R. Oliveira and Wanda Viegas

***Correspondence**: **Filipa Monteiro** fmonteiro@isa.ulisboa.pt

Supplementary Table S5. Pairwise genetic distances between all populations. Cavalli-Sforza and Edward's chord distance (DC^{INA}, upper-right matrix) and Nei's D distance (lower-left matrix).

Cavalli-Sforza and Edward's chord distance (DC^{INA})

Nei's Distance (*D*) (rows)

	Aile	Anton	Dankow	Imperial	Kungs	Petkus	Voima	Pulaw	Alvao	Riodeva	Sved	R2136Russ	R780Spain	R2694West	R1148Turk	R1138Ital	R1133PT	SECCE1	SECCE2	SECCE3	SECCE4	SECCE5	SECCE6	SECCE7	SECCE8	SECCE9	SECCE10	SECCE11	*S. strictum*
Aile	-	0,41	0,45	0,48	0,46	0,41	0,45	0,42	0,36	0,48	0,48	0,42	0,46	0,43	0,41	0,42	0,48	0,48	0,52	0,50	0,47	0,49	0,47	0,46	0,44	0,49	0,49	0,44	0,58
Anton	0,39	-	0,39	0,37	0,43	0,41	0,42	0,39	0,39	0,44	0,38	0,36	0,41	0,34	0,39	0,43	0,44	0,50	0,53	0,48	0,51	0,49	0,44	0,45	0,43	0,42	0,45	0,38	0,54
Dankow	0,42	0,29	-	0,42	0,42	0,32	0,42	0,33	0,42	0,55	0,51	0,43	0,45	0,39	0,43	0,40	0,40	0,44	0,53	0,47	0,52	0,53	0,46	0,46	0,46	0,48	0,47	0,39	0,64
Imperial	0,44	0,27	0,30	-	0,45	0,41	0,48	0,38	0,45	0,54	0,47	0,41	0,39	0,45	0,44	0,36	0,40	0,49	0,54	0,50	0,53	0,51	0,49	0,49	0,48	0,50	0,46	0,43	0,58
Kungs	0,48	0,57	0,31	0,54	-	0,35	0,33	0,44	0,44	0,57	0,48	0,42	0,39	0,36	0,47	0,45	0,49	0,51	0,53	0,50	0,56	0,51	0,47	0,48	0,47	0,49	0,48	0,41	0,64
Petkus	0,40	0,42	0,17	0,38	0,23	-	0,38	0,38	0,39	0,57	0,47	0,40	0,44	0,37	0,39	0,38	0,45	0,43	0,51	0,48	0,54	0,52	0,46	0,47	0,46	0,46	0,47	0,37	0,61
Voima	0,39	0,31	0,27	0,38	0,24	0,27	-	0,47	0,41	0,53	0,45	0,44	0,39	0,35	0,47	0,42	0,44	0,47	0,51	0,49	0,53	0,46	0,46	0,45	0,42	0,45	0,47	0,37	0,54
Pulaw	0,38	0,26	0,16	0,27	0,39	0,29	0,33	-	0,44	0,54	0,50	0,42	0,46	0,44	0,43	0,41	0,41	0,49	0,54	0,49	0,52	0,52	0,48	0,48	0,47	0,50	0,48	0,42	0,56
Alvao	0,23	0,28	0,26	0,33	0,42	0,28	0,25	0,29	-	0,53	0,47	0,41	0,41	0,40	0,38	0,39	0,42	0,41	0,49	0,46	0,49	0,47	0,44	0,46	0,40	0,45	0,44	0,38	0,58
Riodeva	0,43	0,40	0,69	0,60	0,81	0,75	0,55	0,60	0,49	-	0,32	0,48	0,49	0,42	0,50	0,51	0,51	0,54	0,54	0,55	0,50	0,47	0,47	0,38	0,43	0,38	0,42	0,37	0,48
Sved	0,48	0,28	0,62	0,50	0,59	0,53	0,46	0,53	0,48	0,21	-	0,44	0,44	0,43	0,46	0,48	0,46	0,49	0,52	0,50	0,53	0,45	0,44	0,36	0,38	0,32	0,42	0,30	0,50
R2136Russ	0,39	0,22	0,36	0,36	0,40	0,38	0,26	0,31	0,29	0,39	0,35	-	0,40	0,35	0,42	0,41	0,49	0,48	0,48	0,44	0,48	0,48	0,43	0,47	0,43	0,47	0,44	0,41	0,53
R780Spain	0,37	0,33	0,33	0,32	0,40	0,41	0,26	0,35	0,24	0,45	0,43	0,31	-	0,37	0,44	0,41	0,36	0,46	0,51	0,47	0,51	0,49	0,44	0,46	0,46	0,45	0,41	0,37	0,58
R2694West	0,42	0,25	0,31	0,38	0,40	0,33	0,17	0,36	0,31	0,43	0,42	0,21	0,29	-	0,41	0,38	0,42	0,46	0,51	0,49	0,53	0,50	0,45	0,44	0,40	0,44	0,42	0,37	0,52
R1148Turk	0,29	0,22	0,37	0,33	0,57	0,34	0,32	0,34	0,25	0,36	0,38	0,31	0,32	0,29	-	0,40	0,41	0,40	0,45	0,46	0,48	0,47	0,42	0,43	0,44	0,46	0,41	0,38	0,56
R1138Ital	0,30	0,41	0,33	0,33	0,47	0,28	0,30	0,32	0,25	0,45	0,47	0,39	0,35	0,26	0,25	-	0,41	0,43	0,51	0,49	0,50	0,49	0,44	0,46	0,45	0,49	0,45	0,40	0,58
R1133PT	0,34	0,33	0,29	0,31	0,61	0,38	0,31	0,32	0,27	0,42	0,43	0,43	0,20	0,34	0,25	0,27	-	0,43	0,50	0,50	0,50	0,49	0,47	0,45	0,46	0,41	0,42	0,36	0,61
SECCE1	0,39	0,54	0,49	0,50	0,60	0,41	0,40	0,53	0,34	0,40	0,51	0,44	0,40	0,44	0,33	0,39	0,35	-	0,36	0,35	0,39	0,38	0,37	0,43	0,38	0,43	0,37	0,36	0,61
SECCE2	0,44	0,49	0,65	0,53	0,64	0,50	0,41	0,62	0,42	0,40	0,50	0,39	0,49	0,48	0,31	0,47	0,47	0,18	-	0,32	0,33	0,34	0,35	0,40	0,41	0,42	0,39	0,42	0,56
SECCE3	0,34	0,35	0,43	0,37	0,45	0,40	0,34	0,45	0,33	0,42	0,44	0,26	0,32	0,35	0,31	0,40	0,43	0,20	0,15	-	0,33	0,35	0,34	0,42	0,41	0,45	0,39	0,43	0,56
SECCE4	0,35	0,45	0,62	0,49	0,70	0,59	0,47	0,51	0,37	0,33	0,48	0,36	0,43	0,50	0,34	0,47	0,38	0,23	0,13	0,16	-	0,33	0,33	0,39	0,38	0,42	0,39	0,42	0,54
SECCE5	0,37	0,44	0,63	0,45	0,63	0,55	0,39	0,51	0,33	0,21	0,30	0,38	0,36	0,45	0,32	0,39	0,35	0,20	0,17	0,18	0,12	-	0,31	0,32	0,34	0,32	0,39	0,39	0,52
SECCE6	0,33	0,31	0,51	0,48	0,55	0,45	0,35	0,46	0,29	0,28	0,32	0,34	0,31	0,34	0,23	0,32	0,34	0,21	0,20	0,18	0,15	0,14	-	0,32	0,35	0,37	0,37	0,35	0,57
SECCE7	0,32	0,31	0,47	0,43	0,53	0,45	0,36	0,43	0,32	0,15	0,17	0,36	0,33	0,35	0,25	0,31	0,29	0,29	0,29	0,28	0,22	0,13	0,14	-	0,27	0,26	0,32	0,27	0,53
SECCE8	0,27	0,24	0,42	0,37	0,51	0,42	0,27	0,35	0,24	0,18	0,18	0,31	0,28	0,25	0,22	0,32	0,26	0,26	0,29	0,28	0,24	0,15	0,17	0,08	-	0,31	0,34	0,31	0,51
SECCE9	0,41	0,29	0,51	0,45	0,63	0,48	0,38	0,47	0,35	0,16	0,17	0,37	0,32	0,37	0,28	0,39	0,24	0,27	0,28	0,27	0,22	0,15	0,18	0,08	0,10	-	0,35	0,29	0,52
SECCE10	0,36	0,30	0,45	0,38	0,54	0,42	0,36	0,41	0,30	0,24	0,29	0,28	0,26	0,34	0,22	0,38	0,23	0,22	0,24	0,28	0,23	0,23	0,22	0,14	0,15	0,14	-	0,27	0,52
SECCE11	0,34	0,25	0,32	0,35	0,44	0,31	0,27	0,33	0,24	0,20	0,16	0,32	0,25	0,28	0,24	0,31	0,21	0,26	0,34	0,33	0,29	0,22	0,20	0,09	0,10	0,10	0,12	-	0,53
S. strictum	0,80	0,56	0,98	0,70	1,11	0,89	0,65	0,70	0,73	0,57	0,56	0,57	0,75	0,63	0,58	0,74	0,71	0,75	0,55	0,51	0,56	0,46	0,63	0,58	0,47	0,48	0,59	0,64	-
