# Supplementary material for: Genetic Distinctiveness of Rye In situ Accessions from Portugal Unveils a New Hotspot of Unexplored Genetic Resources
Source: Front Plant Sci. 2016 Aug 31;7:1334. doi: 10.3389/fpls.2016.01334 (PMC5006150; doi:10.3389/fpls.2016.01334)
Supplement: Supplementary file 7 [file Image1.pdf]

Supplementary Material

Genetic distinctiveness of rye *in situ* accessions from Portugal unveils a new hotspot of unexplored genetic resources

Filipa Monteiro*, Patrícia Vidigal, André B. Barros, Ana Monteiro, Hugo R. Oliveira and Wanda Viegas

***Correspondence**: **Filipa Monteiro** fmonteiro@isa.ulisboa.pt

Supplementary Figure S1. UPGMA (A) and NJ (B) trees generated from Cavalli-Sforza and Edward's chord distance (*DC* ^{INA}). Accessions are indicated by symbols reflecting grouping assignment: ■ cultivars, ⭘ *ex situ* accessions, ● *in situ* accessions. *S. strictum* was used as a species outgroup (➤). Only bootstraps values above 50 are indicated.

A

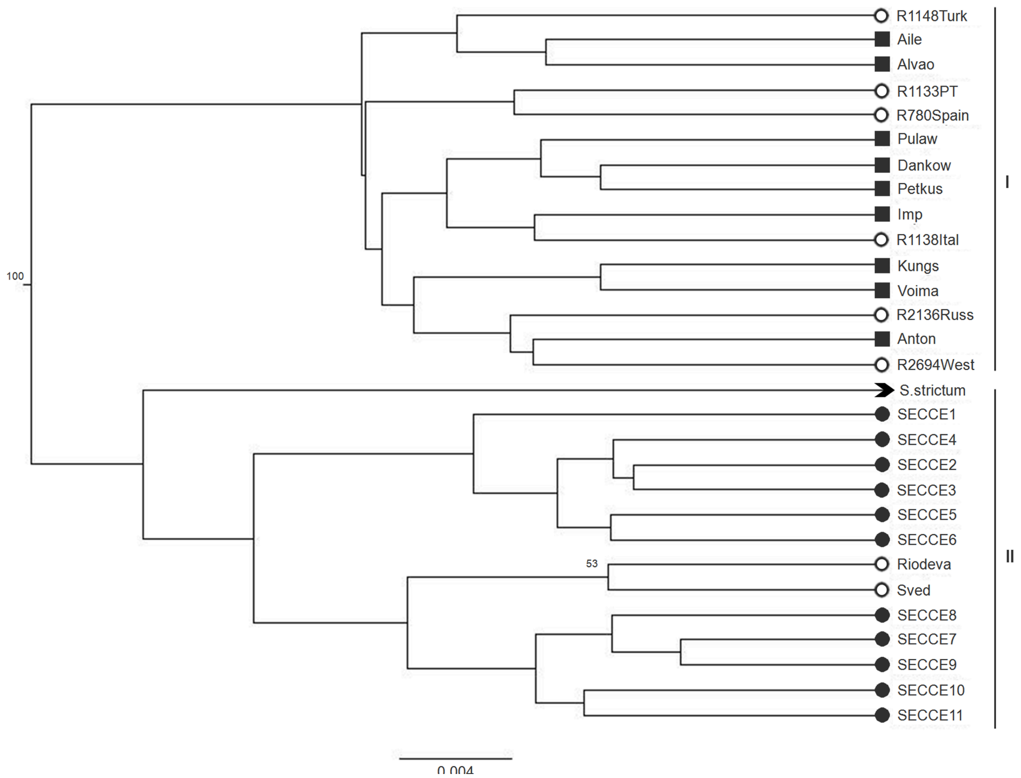

B

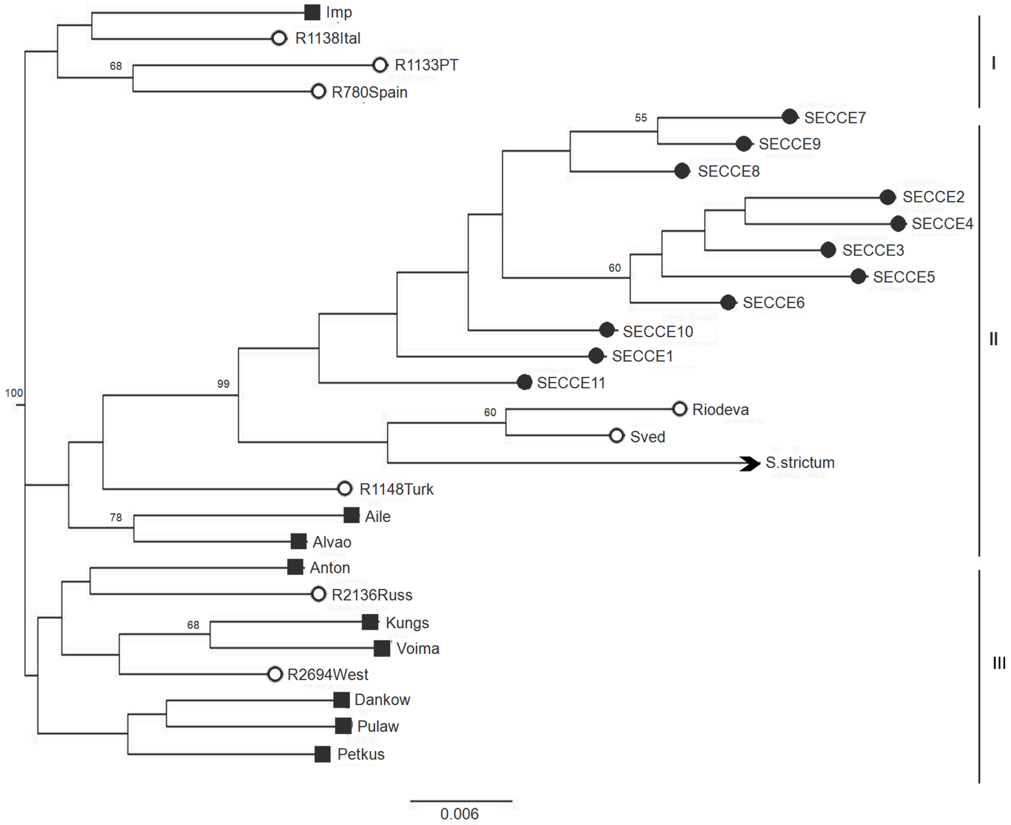
Imp
R1138Ital
68
R1133PT
R780Spain
55
SECCE7
SECCE9
SECCE8
SECCE2
SECCE4
SECCE3
60
SECCE5
SECCE6
SECCE10
SECCE1
SECCE11
99
100
60
Riodeva
Sved
S.strictum
R1148Turk
78
Aile
Alvao
Anton
R2136Russ
68
Kungs
Voima
R2694West
Dankow
Pulaw
Petkus
I
II
III
0.006
