# Supplementary material for: Genetic Distinctiveness of Rye In situ Accessions from Portugal Unveils a New Hotspot of Unexplored Genetic Resources
Source: Front Plant Sci. 2016 Aug 31;7:1334. doi: 10.3389/fpls.2016.01334 (PMC5006150; doi:10.3389/fpls.2016.01334)

Supplementary Material

Genetic distinctiveness of rye *in situ* accessions from Portugal unveils a new hotspot of unexplored genetic resources

Filipa Monteiro*, Patrícia Vidigal, André B. Barros, Ana Monteiro, Hugo R. Oliveira and Wanda Viegas

***Correspondence**: **Filipa Monteiro** fmonteiro@isa.ulisboa.pt

Supplementary Figure S2. STRUCTURE *ad hoc* statistics retrieved by StructureHarvester using 1 to 10 possible clusters (*K*). (**A**) Variation of the posterior log-probability of the data as a function of the number of clusters K; (**B**) Variation of Δ*K* values according to Evanno *et al.* (2005) method.

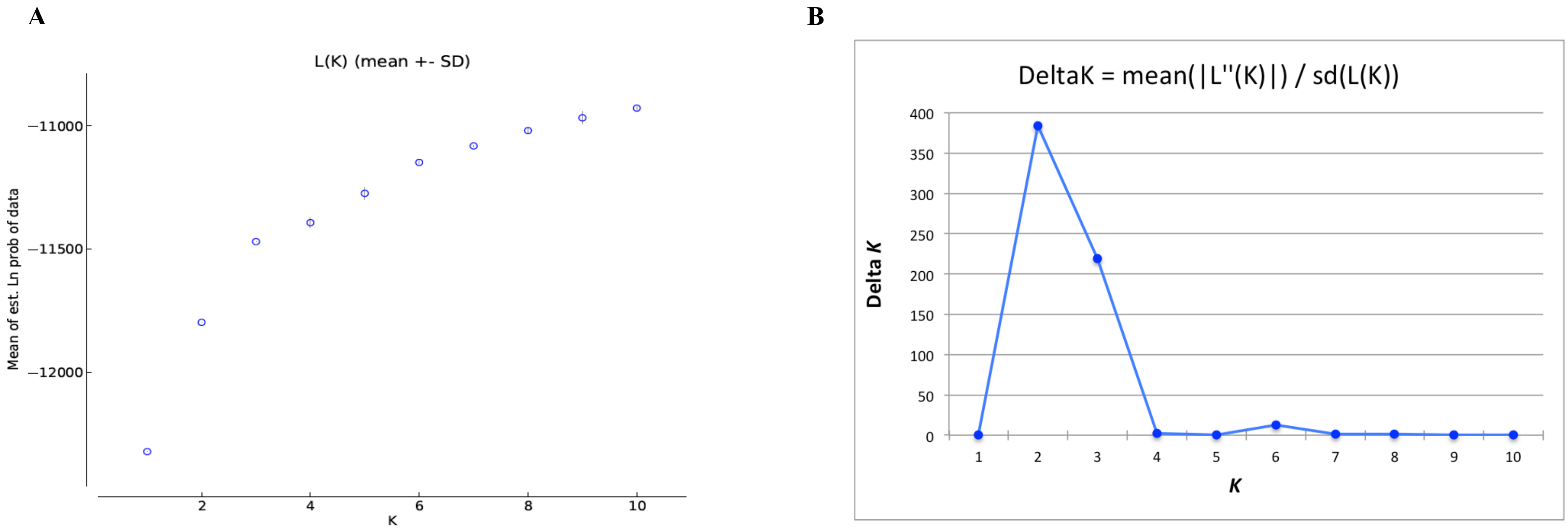

Supplement: Supplementary file 8 [file Image2.pdf]
