# Supplementary material for: Genetic Distinctiveness of Rye In situ Accessions from Portugal Unveils a New Hotspot of Unexplored Genetic Resources
Source: Front Plant Sci. 2016 Aug 31;7:1334. doi: 10.3389/fpls.2016.01334 (PMC5006150; doi:10.3389/fpls.2016.01334)

Supplementary Material

Genetic distinctiveness of rye *in situ* accessions from Portugal unveils a new hotspot of unexplored genetic resources

Filipa Monteiro*, Patrícia Vidigal, André B. Barros, Ana Monteiro, Hugo R. Oliveira and Wanda Viegas

***Correspondence**: **Filipa Monteiro** fmonteiro@isa.ulisboa.pt

Supplementary Figure S3. DAPC results. (1) Inference of the number of clusters using DAPC *find.clusters* function and **(2)** Loading plots of the two Discriminant Functions following DAPC analysis with a *K*=3. **(2)** Loading plots of the two Discriminant Functions following DAPC analysis with a *K*=3.

1. Inference of the number of clusters using DAPC *find.clusters* function. A *K* value of 3 (the lowest BIC value) represents the best summary of the data.

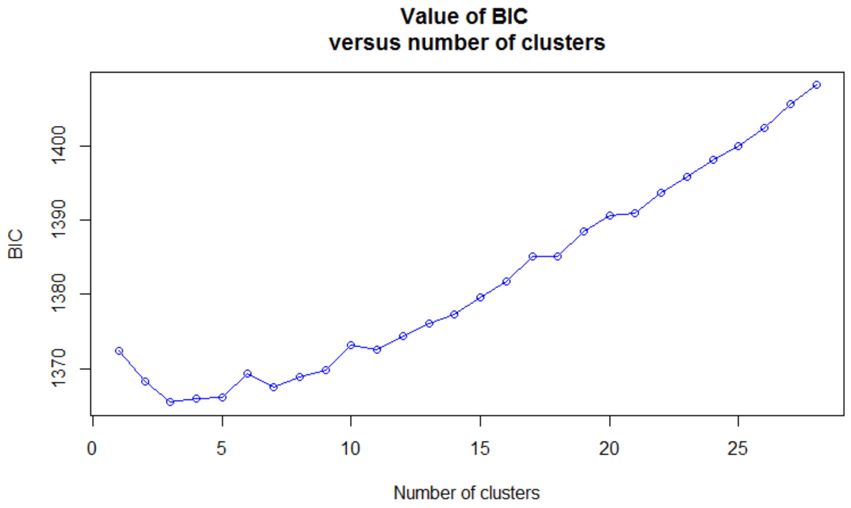

2. Loading plots of the two Discriminant Functions following DAPC analysis with a *K*=3. (**A**) Allele loading plots from DF1 and (**B**) from DF2, after assigning a 0.045 as threshold.

A

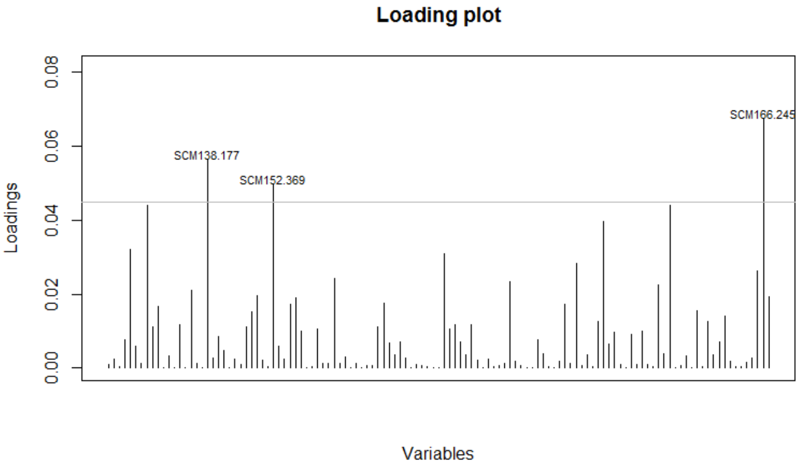

B

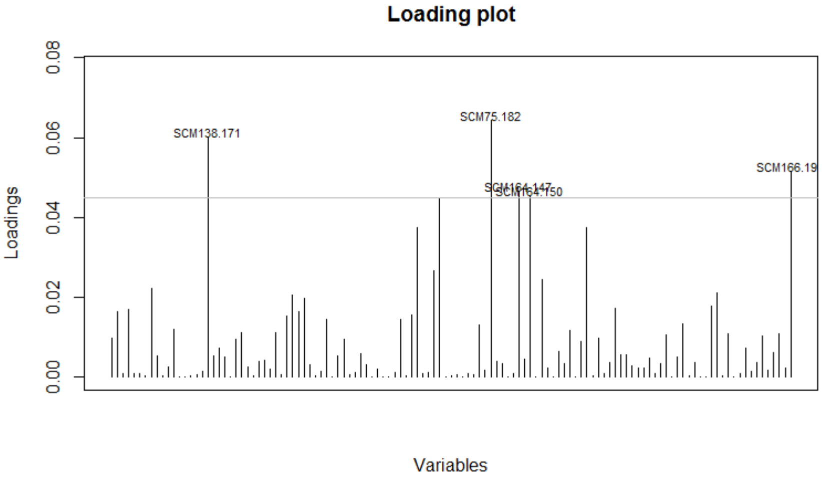

Supplement: Supplementary file 9 [file Image3.pdf]
