# Supplementary material for: Genetic Distinctiveness of Rye In situ Accessions from Portugal Unveils a New Hotspot of Unexplored Genetic Resources
Source: Front Plant Sci. 2016 Aug 31;7:1334. doi: 10.3389/fpls.2016.01334 (PMC5006150; doi:10.3389/fpls.2016.01334)

Supplementary Material

Genetic distinctiveness of rye *in situ* accessions from Portugal unveils a new hotspot of unexplored genetic resources

Filipa Monteiro*, Patrícia Vidigal, André B. Barros, Ana Monteiro, Hugo R. Oliveira and Wanda Viegas

***Correspondence**: **Filipa Monteiro** fmonteiro@isa.ulisboa.pt

Supplementary Figure S4. Scatterplot of DAPC for *K*=3 assignment. Scatterplot shows the two principal components of the DAPC and clusters are numbered and displayed by different colours, while dots represent individuals. The 2 Discriminant Functions hereby represented explain 93% of cumulative variance of the dataset.

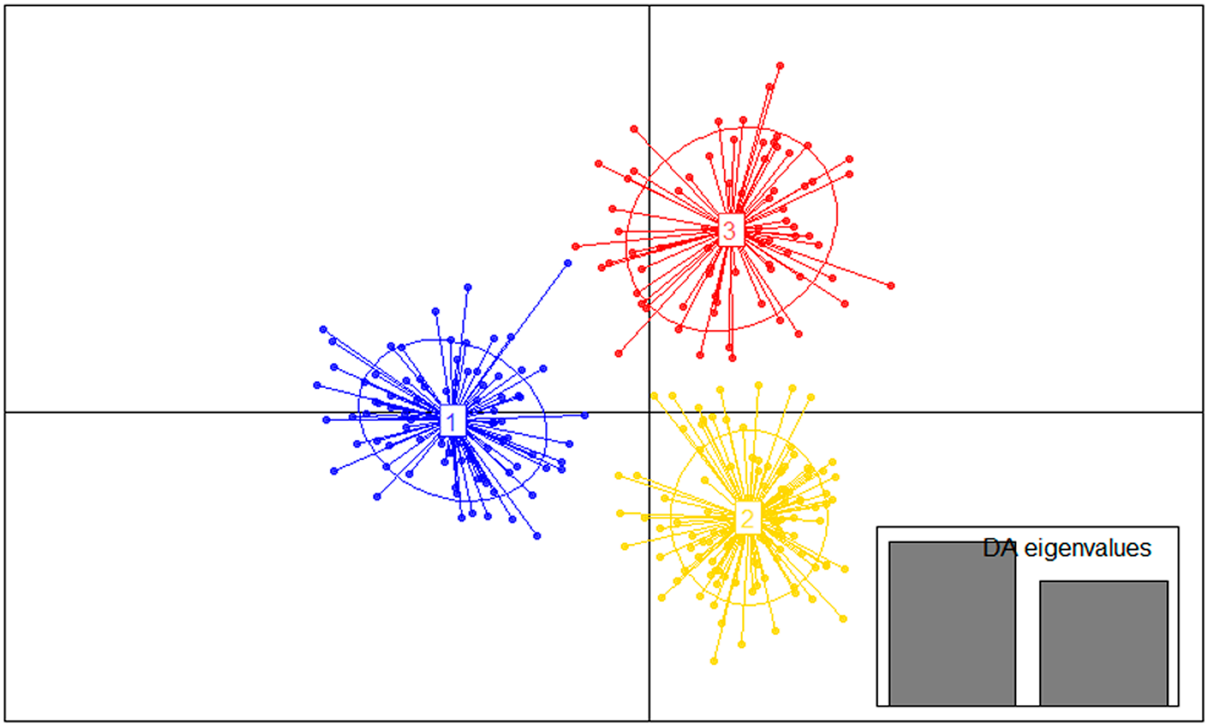

Supplement: Supplementary file 10 [file Image4.pdf]
